# White matter associations with spelling performance

**DOI:** 10.1007/s00429-024-02775-7

**Published:** 2024-03-25

**Authors:** Romi Sagi, J. S. H. Taylor, Kyriaki Neophytou, Tamar Cohen, Brenda Rapp, Kathleen Rastle, Michal Ben-Shachar

**Affiliations:** 1 The Gonda Multidisciplinary Brain Research Center, Bar-Ilan University, Ramat-Gan, Israel; 2 Division of Psychology and Language Sciences, University College London, London, UK; 3 Department of Cognitive Science, Johns Hopkins University, Baltimore, USA; 4 Department of Psychology, Royal Holloway, University of London, London, UK; 5 Department of Neurology, Johns Hopkins Medicine, Baltimore, USA

**Keywords:** Spelling, White matter, DTI, Diffusion MRI, Probabilistic tractography, Written word production

## Abstract

Multiple neurocognitive processes are involved in the highly complex task of producing written words. Yet, little is known about the neural pathways that support spelling in healthy adults. We assessed the associations between performance on a difficult spelling-to-dictation task and microstructural properties of language-related white matter pathways, in a sample of 73 native English-speaking neurotypical adults. Participants completed a diffusion magnetic resonance imaging scan and a cognitive assessment battery. Using constrained spherical deconvolution modeling and probabilistic tractography, we reconstructed dorsal and ventral white matter tracts of interest, bilaterally, in individual participants. Spelling associations were found in both dorsal and ventral stream pathways. In high-performing spellers, spelling scores significantly correlated with fractional anisotropy (FA) within the left inferior longitudinal fasciculus, a ventral stream pathway. In low-performing spellers, spelling scores significantly correlated with FA within the third branch of the right superior longitudinal fasciculus, a dorsal pathway. An automated analysis of spelling errors revealed that high- and low- performing spellers also differed in their error patterns, diverging primarily in terms of the orthographic distance between their errors and the correct spelling, compared to the phonological plausibility of their spelling responses. The results demonstrate the complexity of the neurocognitive architecture of spelling. The distinct white matter associations and error patterns detected in low- and high- performing spellers suggest that they rely on different cognitive processes, such that high-performing spellers rely more on lexical-orthographic representations, while low-performing spellers rely more on phoneme-to-grapheme conversion.

**Supplementary Information:**

The online version contains supplementary material available at 10.1007/s00429-024-02775-7.

## Introduction

In the modern world, written language production is used daily as an essential part of communication, including activities such as emailing or texting. Producing written words relies on a highly complex neurocognitive system, which involves mentally retrieving or generating orthographic representations and temporarily maintaining the identity and order of letter sequences while planning and executing the corresponding motor output. However, compared to other language modalities such as reading or speaking, spelling remains a relatively understudied domain, particularly in terms of its neural bases. Proficient spelling requires efficient communication within a distributed cortical network of frontal, parietal, temporal, and occipitotemporal brain regions (Planton et al. 2013; Purcell et al. 2011b). Therefore, it likely relies on the neuroanatomical connections that transmit information between these regions. Yet, little is known about the white matter pathways that support spelling. Our goal here is to identify white matter pathways that support spelling in neurotypical adults, by evaluating the associations between spelling performance and microstructural tract properties assessed in the same individuals.

Cognitive models of spelling have been developed primarily based on the performance patterns of individuals with acquired dysgraphia (e.g., Hillis et al. 2004; Rapp and Caramazza 1997; Roeltgen and Heilman 1984, 1985). These models generally describe two routes for spelling a word: lexical and sublexical (Baxter and Warrington 1985; Beauvois and Dérouesené 1981; Goodman-Schulman and Caramazza 1987; Shallice 1981). Spelling via the lexical route involves retrieving an orthographic word-form from the orthographic lexicon (also referred to as the orthographic long-term memory). This word-form is then held in orthographic working memory (also known as the graphemic buffer) while planning the motor movements to produce the written output (e.g., handwriting, typing, etc.). The lexical route provides an efficient approach for spelling familiar words. The sublexical route relies on converting speech sounds, phonemes, into letter representations (graphemes) to assemble plausible spellings of orthographically regular or unfamiliar words, including pseudowords. For example, spelling the irregular word ‘laugh’ as LAF indicates reliance on phoneme-to-grapheme conversion via the sublexical route rather than on lexical-orthographic retrieval via the lexical route. This type of spelling error, which may be orthographically distant from the correct spelling, is defined as a phonologically plausible error (Goodman and Caramazza 1986b).

The neurocognitive dynamics of lexical and sublexical processes in spelling is not well understood. Evidence from individuals with dysgraphia indicates that spelling is not necessarily generated by one route or the other, but often involves integration and competition between lexical and sublexical processes (Folk et al. 2002; Folk and Rapp 2004; McCloskey et al. 2006; Rapp et al. 2002). For example, spelling ‘bouquet’ as BOUKET indicates involvement of both sublexical and lexical processes (sublexical: the sound/k/spelled as K instead of QU, lexical:/ei/spelled as ET; Rapp et al. 2002). These issues have been addressed by connectionist models of spelling. For example, a dual-route connectionist model proposed by Houghton and Zorzi (2003) suggests that in typical spellers, both routes are activated in parallel, with competitive-cooperative interactions, and their combined output determines the final spelling (for other distributed implementations of spelling, see Brown and Loosemore 1995; Bullinaria 1994; Loosemore et al. 1991). To provide a deeper understanding of the reliance on lexical and sublexical spelling processes in neurotypical individuals, we examined the characteristics of the spelling errors produced by participants, specifically evaluating their phonological plausibility and their lexical-orthographic distance from the correct spelling.

A small body of functional neuroimaging studies examined the cortical regions that support spelling in neurotypical individuals (Planton et al. 2013; Purcell et al. 2011b). Across studies, a distributed network of areas has been identified as being reliably responsive during spelling tasks, including regions within both the dorsal and ventral language streams (as defined by Hickok and Poeppel 2007; Rauschecker 2012). Dorsally, multiple frontal, parietal, and temporal areas have been identified, including the left inferior and middle frontal gyrus, bilateral superior frontal sulcus, left supplementary motor area, left superior and bilateral inferior parietal lobules, and bilateral posterior superior temporal cortex. Ventrally, activated regions included the inferior temporal gyrus and left posterior fusiform gyrus. Specifically, the visual word form area (VWFA), which has been linked to the orthographic lexicon (Glezer et al. 2009; Lerma-Usabiaga et al. 2018; Rapp and Caramazza 1997; Rapp and Lipka 2011; Thesen et al. 2012), has been consistently associated with central spelling processes (e.g., orthographic long-term memory) rather than peripheral spelling processes (e.g., motor programming) (Ludersdorfer et al. 2015; Purcell et al. 2011b). On the whole, prior studies suggest that spelling requires coordinated processing across multiple distributed brain areas. Therefore, white matter pathways connecting those regions are of particular interest in understanding the neurobiology of spelling.

Studying the microstructural properties of white matter in the living human brain became possible in the last two decades, using diffusion MRI. While numerous diffusion magnetic resonance imaging (dMRI) studies have been conducted on written word recognition (Ben-Shachar et al. 2007; Vandermosten et al. 2012b; Wandell and Le 2017; Wandell and Yeatman 2013), only a few studies have examined white matter structures related to written word production, focusing primarily on spelling impairments. Following left-hemisphere ischemic stroke, impaired maintenance of letter sequences in working memory during spelling was associated with ischemia in white matter adjacent to the prefrontal cortex, lateral occipital gyrus, or caudate (Cloutman et al. 2009). Spelling-impaired children showed altered dMRI parameters at the voxel level mainly in right dorsal white matter, including the right superior corona radiata, right posterior limb of the internal capsule, and the right superior longitudinal fasciculus (SLF) (Gebauer et al. 2012). In children with a specific spelling deficit, spelling abilities were associated with microstructural properties within the left arcuate fasciculus, while in dyslexic children spelling performance correlated with properties of the bilateral inferior longitudinal fasciculus (ILF), right SLF, and left cingulum (Banfi et al. 2019). In sum, prior studies of white matter in impaired populations report associations with spelling abilities in both dorsal and ventral white matter pathways. To our knowledge, only one recent study examined spelling-related white matter in neurotypical adults, reporting that spelling performance was associated with the left ILF in skilled readers and the uncinate fasciculus in impaired readers (Cheema et al. 2022).

The current study examines the associations between spelling performance among neurotypical adults and specific white matter tracts hypothesized to support spelling processes. We construct our hypotheses based on converging evidence from neuroimaging studies of written word production (Banfi et al. 2019; Cheema et al. 2022; Gebauer et al. 2012; Planton et al. 2013; Purcell et al. 2011b) and lesion data from brain-damaged patients with acquired dysgraphia (Alexander et al. 1992; Beauvois and Dérouesené, 1981; Bub and Kertesz 1982; Caramazza et al. 1987; Goodman and Caramazza 1986b, 1986a; Henry et al. 2007; Hillis et al. 2004; Rapcsak et al. 1988; Rapcsak and Beeson 2004; Rapp et al. 2016; Roeltgen and Heilman 1984, 1985; Shallice 1981; Tomasino et al. 2015). Within the framework of the dual-stream model for the functional anatomy of language (Hickok and Poeppel 2007), since spelling involves both lexical and sublexical processes, we hypothesize that spelling abilities could be associated with both lexically-related ventral tracts and phonologically-related dorsal tracts. Dorsally, as spelling has been consistently associated with parietal and frontal regions (e.g., Purcell et al. 2011a), the frontoparietal SLF is a primary candidate tract to convey sublexical spelling information. Additionally, the frontotemporal arcuate fasciculus is also identified as a dorsal candidate tract, due to spelling associations with superior temporal areas in addition to the left inferior frontal gyrus (e.g., Booth et al. 2002). Ventrally, based on reliable spelling associations with the VWFA (e.g., Rapp and Dufor 2011,2011), the ILF, which connects the VWFA with ventral anterior temporal regions, is hypothesized to be involved in lexical processes in spelling.

Seventy-three neurotypical English-speaking adults were tested on a difficult spelling-to-dictation task designed to obtain substantial variability in spelling accuracy within typical adult spellers. In addition to evaluating spelling accuracy among participants, we characterize the patterns of their spelling errors, by calculating the orthographic and phonological distance of errors from the target spellings. All participants underwent an MRI scan, including diffusion and T1 MRI sequences. We use constrained spherical deconvolution (CSD) modeling, coupled with probabilistic tractography, to reconstruct the bilateral dorsal and ventral language-related white-matter pathways of interest in each participant. To date, most dMRI studies analyze the SLF as one complex. However, post-mortem dissections and in vivo dMRI studies in humans suggest that, similarly to monkeys, the SLF is composed of three branches: SLF-I (dorsal), SLF-II (middle), and SLF-III (ventral) (Amemiya et al. 2021; Makris et al. 2005; Petrides and Pandya 1984; Schmahmann and Pandya 2007; Schurr et al. 2020; Thiebaut de Schotten et al. 2011b; Thiebaut de Schotten et al. 2012), which are hypothesized to vary in their functional involvement in cognitive processes (Makris et al. 2005). Here, we extend existing automatic segmentation tools (Yeatman et al. 2012b) to segment the SLF into three components, and examine the distinct associations of each of the three SLF branches, the arcuate fasciculus, and the ILF, with spelling performance.

## Materials and methods

### Participants

The sample included 73 native English-speaking adult participants recruited at the Royal Holloway, University of London (57 females, mean age 21y, *SD* = 2.7, age range 19–35; the age of one participant was recorded incorrectly and therefore excluded from the age data). All participants were right-handed and had no history of diagnosed learning disabilities or neurological conditions. Participants underwent an extensive cognitive assessment and MRI scans as part of a larger research project. Some of these participants were included in previously published studies reporting behavioral, functional- and diffusion- MRI measurements (Rastle et al. 2021, n = 48; Taylor et al. 2017; 2019, n = 24; Yablonski et al. 2019, n = 45). None of these studies analyzed the spelling data and none reported data from the entire sample included here. See also “Ethics approval” and “Consent to participate” statements under Declarations.

### Cognitive assessment

The entire cognitive assessment was conducted in a quiet room and lasted approximately one hour. In the current analysis, we focused on the spelling task (“Spelling task”), and included a few additional tests to control for cognitive components related to the cognitive architecture of spelling (“Additional cognitive measures”).

#### Spelling task

Participants completed a spelling-to-dictation task comprised of 40 English words. To obtain a range of spelling performance and avoid a ceiling effect, stimuli consisted of long, low-frequency words (8–10 letters) with one-to-many phoneme to grapheme mappings (see Table S1 for a complete list of stimuli). These words were selected from a larger pool of items tested in a previous study of spelling-to-dictation and lexical decision (Burt and Tate 2002), with new carrier sentences created to provide contextual information about each word. Previous studies using this test have yielded substantial variation in spelling scores (Ulicheva et al. 2021a, b). On each trial, participants heard a recorded word, first presented in isolation and then as part of the spoken carrier sentence (e.g., “Dissuade – His friends tried to dissuade him from flying”). Auditory stimuli were recorded by a native female speaker of Southern British English and presented via headphones. Participants were asked to type the target word on a standard QWERTY keyboard (they could use backspace to correct any mistakes), then press ENTER to move on to the subsequent trial, with no time limit for each response.

#### Additional cognitive measures

*Vocabulary* To evaluate lexical-semantic knowledge, participants were asked to select which of four alternatives was closest in meaning to a given target word. The proportion of correct items out of 40 trials was calculated (Shipley 1940). *Speeded word and pseudoword reading* To evaluate word reading and phonemic decoding, participants completed the Test of Word Reading Efficiency second edition (TOWRE-2; Torgesen et al. 2012), which consists of two subtests: sight word efficiency (SWE) and phonemic decoding efficiency (PDE). SWE assesses the number of words read aloud correctly in 45 s (out of a list of 108 words). PDE assesses the number of pseudowords read aloud correctly in 45 s (out of a list of 66 pseudowords). The final scores were scaled according to the norms of the appropriate age group (17—24 years). *Spoonerisms* To assess phonological awareness, participants listened to word pairs and were asked to repeat each pair while swapping the initial phonemes of the two words (e.g., chestnut-people → *pestnut*-*cheople*). The test included 20 word pairs from the Phonological Assessment Battery (Frederickson et al. 1997), and the proportion of correct trials was calculated. *Nonword repetition* Assessed using a subtest of the Comprehensive Test of Phonological Processing (CToPP-2; Wagner et al. 1999). In each trial, participants listened to a single pseudoword and were asked to repeat it. The test included 30 items, gradually increasing in length. The number of correct responses was recorded and scaled according to age norms. *Rapid Automatized Naming* (RAN, subtest of CToPP-2; Wagner et al. 1999): Participants were asked to quickly name letters organized in a matrix, while measuring the time to complete the naming of the entire matrix.

### Behavioral analyses

#### Spelling accuracy scores

For each participant, a total spelling accuracy score between 0 and 1 was calculated as the proportion of correctly spelled items out of a total of 40 test items. Responses that contained any inaccuracy compared with the correct spelling of the target were considered incorrect. To evaluate the associations between spelling accuracy scores and other cognitive measures, Spearman’s pairwise correlation coefficients were calculated between every pair of measures. Multiple comparisons across all 21 pairs of behavioral tests were handled by controlling the false discovery rate (FDR; Benjamini and Hochberg 1995) at a level of q < 0.05, such that only correlations that passed this criterion are considered significant. For consistency with the direction of all other measures (higher score—better performance), we multiplied RAN results by −1 before calculating correlations with other cognitive measures. We used Spearman's correlations because the distribution of spelling scores did not satisfy the normality criterion (as indicated by a Shapiro–Wilk test, see “Results”, Sect. "Spelling accuracy scores").

#### Orthographic and phonological distance

To further characterize the types of spelling errors produced by the participants, each response was assessed by calculating the orthographic and phonological distances of the response from the target spelling (Themistocleous et al. 2020). The *orthographic distance* was used to evaluate the orthographic deviation of an error from the actual spelling of the target. It was calculated as the normalized Damerau-Levenshtein distance between the response and the target, which identifies the minimal number of operations required to make the two letter strings identical. Specifically, it quantifies the number of letter insertions, deletions, substitutions or transpositions of letters, divided by the number of characters of the target or the response (whichever is longer, following the procedure in Themistocleous et al. 2020, to produce a distance measure bound at 1). The *phonological distance* was used to evaluate the extent of the phonological implausibility of the response. It measures the deviation between the phonological form of the response and the phonological form of the target. First, both the target and the response were transcribed into the International Phonetic Alphabet (IPA), then the distance between the resulting phonetic transcriptions was calculated using the normalized Damerau-Levenshtein distance (as described above). Phonetic transcriptions were generated using eSpeak, a text-to-speech open-source software (Duddington and Dunn 2012). Both the orthographic and the phonological distance for a given response could range between 0 (for an accurate response) and 1. For example, spelling ‘dissuade’ as DISWAYED yields an orthographic distance of 0.5 and a phonological distance of 0, whereas spelling the same word as DISSAUDE yields an orthographic distance of 0.125 and a phonological distance of 0.43.

### MRI data acquisition

MRI scans were collected on a 3T Siemens Trio scanner (Siemens Medical Systems, Erlangen, Germany) with a 32-channel head coil. The MRI protocol included T1 and diffusion imaging sequences, as detailed below.

#### T1 image acquisition

High-resolution T1-weighted anatomical images were acquired for each participant using a magnetization-prepared rapid acquisition gradient echo (MPRAGE) protocol (TR = 2250 ms, TE = 2.99 ms, flip angle = 9°, 1 mm thick slices, voxel size: 1 × 1 × 1 mm).

#### Diffusion-weighted image acquisition

A standard dMRI protocol was applied using a single-shot spin-echo diffusion-weighted echo-planar imaging (DW-EPI) sequence (63 axial slices, each 2 mm thick with no gap; field of view = 192 × 192 mm, image matrix size = 96 × 96 providing a cubic resolution of 2 × 2 × 2 mm). 64 diffusion-weighted volumes (*b* = 1000 s/mm^2^) and one reference volume (*b* = 0 s/mm^2^) were acquired using a standard diffusion direction matrix. The total scan time for the dMRI sequence was 8:52 min.

### White matter analysis

The analysis of dMRI data was performed within the native space of each individual participant, and consisted of three main steps, as detailed below. First, raw diffusion data were preprocessed, and local models of the diffusion directionality were fit at the voxel level (both tensor and CSD modeling). Second, whole-brain probabilistic tractography was performed based on the CSD models. Third, the white matter pathways of interest were segmented using region of interest (ROI)-based automated tools and their microstructural properties were quantified.

#### dMRI preprocessing

dMRI data were preprocessed using ‘mrDiffusion’, an open-source package (https://github.com/vistalab/vistasoft/tree/master/mrDiffusion) implemented in MATLAB 2012b (The MathWorks, Natick, MA). The pipeline included a rigid transformation of the anatomical images to the anterior commissure-posterior commissure (AC-PC) orientation, motion and eddy-current correction of the diffusion-weighted images, alignment of diffusion data to the anatomical data including recalculation of diffusion directions, resampling of diffusion data, and finally, model fit. Specifically, AC and PC were manually identified within each T1 image which was aligned accordingly. DW images were corrected for eddy-current distortions and head motion based on the expected pattern of eddy-current distortions calculated by a constrained non-linear co-registration algorithm (Rohde et al. 2004). Diffusion-weighted volumes were registered to the non-diffusion-weighted (b0) volume, which was registered to the T1 image using a rigid body mutual information maximization algorithm (as implemented in SPM8; Friston and Ashburner 2004). The combined transform resulting from both motion and eddy-current corrections was then applied to the raw diffusion data, maintaining the original voxel size (2 × 2 × 2 mm). These stages correct for image distortions caused by eddy currents and head motion, without a specific correction for susceptibility artifacts. Next, the gradient directions were appropriately adjusted to fit the resampled diffusion data (Leemans and Jones 2009).

#### Diffusion model fitting

We modeled water diffusion at the voxel level using both tensor- and CSD- modeling. The more advanced CSD model was used as the basis to perform probabilistic tractography. The simpler tensor model was used to extract the fractional anisotropy (FA) at each voxel, assessing the extent to which the diffusion is faster in a particular direction over the others. Tensor fitting was carried out in ‘mrDiffusion’ using a standard least-squares algorithm. FA was calculated in each voxel as the normalized standard deviation of the estimated eigenvalues (λ1, λ2, λ3) of the three principal diffusion coefficients (Basser and Pierpaoli 1996).

Whole-brain probabilistic tractography was performed (see “Whole-brain tractography”) based on the CSD models, (Tournier et al. 2004). The CSD model estimates the fiber orientation distribution function (fODF) within each voxel, providing a solution to the crossing fibers challenges that are prevalent across white matter (Tournier et al. 2012). CSD model fitting was performed using the MRtrix3 toolbox (Tournier et al. 2019). To estimate the diffusion response functions, we used the ‘dwi2response’ command and the ‘dhollander’ algorithm (Dhollander et al. 2016; Dhollander and Connelly 2016). fODFs were estimated based on the response functions calculated for white matter and cerebrospinal fluid, using the ‘dwi2fod’ command with the ‘msmt_csd’ algorithm (Jeurissen et al. 2014), based on a CSD model with up to eight spherical harmonics (lmax = 8) (Tournier et al. 2004, 2007).

#### Whole-brain tractography

Probabilistic tractography was performed using the MRtrix3 command ‘tckgen’ with the default ‘iFOD2’ tracking algorithm. A whole-brain white matter mask was generated for each subject from the structural T1 image using the ‘5ttgen’ script, which performs whole-brain segmentation utilizing FSL tools (Smith et al. 2004). Tracking was initiated from 500,000 random seeds within the whole-brain white matter mask. An fODF threshold of 0.1 was used, with a maximum angle of 45° between successive steps and a step size of 0.85 mm. Streamlines were restricted to a minimum length of 50 mm and a maximum length of 200 mm. Streamlines extending beyond the white matter mask were truncated. The whole-brain tractogram was then used to segment the major white matter pathways hypothesized to communicate spelling information.

#### Automatic segmentation of the arcuate fasciculus and ILF

The arcuate fasciculus and the ILF were automatically segmented as described in prior studies (e.g., Yablonski and Ben-Shachar 2020). Tract segmentation was carried out using the Automatic Fiber Quantification package (AFQ; Yeatman et al. 2012b) based on a waypoint ROI approach (Wakana et al. 2007). Specifically, ROIs predefined on the Montreal Neurological Institute (MNI) T2 template were projected to the native space of each individual. Then, the whole-brain tractogram (described in “Whole-brain tractography”) was intersected with the individual ROIs of each tract, using logical ‘AND’ operations, to identify the streamlines that pass through both ROIs. Each tract was segmented bilaterally.

#### Automatic segmentation of the SLF branches

To identify the three SLF branches separately and automatically using AFQ, we defined new ROIs on the MNI T2 template. We then adapted the AFQ algorithm to identify the SLF-I,-II, and -III bilaterally using these newly defined ROIs (ROIs and code are available at https://github.com/yeatmanlab/AFQ/tree/master/SLF123). Each SLF branch was defined by three ROIs, in accordance with Thiebaut de Schotten et al. (2011b): frontal and parietal 'AND' ROIs and a 'NOT' temporal ROI to exclude frontotemporal fibers of the arcuate fasciculus. Frontal ROIs were located on the coronal plane of the AC and defined distinctly for each of the three branches, while the parietal ROI was shared, located on the coronal plane of the PC (see Fig. S1a). CSD-based probabilistic whole-brain tractograms were intersected with these ROIs to define the SLF-I, -II, and -III. This process successfully segmented all three SLF branches in all participants (see Fig. S1b for examples in an individual participant).

Incidentally, in a preliminary inspection, these newly defined ROIs were intersected with whole-brain tractograms generated using tensor-based deterministic tractography (for details on the deterministic tracking procedure see Yablonski et al. 2019). Visual inspection of the resulting deterministic tracts suggested that this process was successful in segmenting the SLF-III, but did not provide sufficiently adequate results for the SLF-I and SLF-II (missing these segments altogether in many individuals and producing tracking errors). The new tools for automatically identifying the SLF segments in AFQ should therefore be used with whole-brain tractograms generated with CSD modeling and probabilistic tracking.

#### Screening of tract segmentation results

An automatic cleaning procedure was applied to the resulting tracts, removing streamlines that were longer than 3 standard deviations from the mean tract length, or that spatially deviated more than 4 standard deviations from the core of each tract. For this purpose, the tract core is defined as the mean of each fiber’s x, y, z coordinates at each node (Yeatman et al. 2012b). Quality assurance of the resulting tracts in each participant’s native space was performed through careful examination of the tractograms by the first and last authors (R.S and M.B-S). Following visual inspection, the parameters for automatic cleaning of the SLF branches were further adjusted to remove streamlines that deviated by 1 standard deviation from the mean tract length (the criterion for spatial deviation remained as before, excluding streamlines that spatially deviated more than 4 standard deviations from the tract core).

#### Lateralization index

To examine the degree of hemispheric asymmetry of each tract, we conducted a lateralization analysis of the five bilateral tracts, following Thiebaut de Schotten et al. (2011a). Specifically, we extracted the number of streamlines (n.streamlines) detected in the right and left homolog tracts and calculated a lateralization index (LI), for each participant, for each of the tracts, as follows:

$${\text{LI}} = \frac{{{\text{Right n}}.\,{\text{streamlines }}-{\text{ Left n}}.\,{\text{streamlines}}}}{{{\text{Right n}}.\,{\text{streamlines }}+{\text{Left n}}.\,{\text{streamlines}}}}$$

A positive LI indicated right lateralization, whereas a negative LI indicated left lateralization. The significance level of the lateralization for each tract was determined using five two-tailed, one-sample t-tests with a Bonferroni-adjusted alpha level of 0.01 per test (0.05/5).

### Spelling-white matter association analyses

Tract quantification was implemented using AFQ (Yeatman et al. 2012b). For each of the tracts of interest, we extracted FA values from 100 equidistant nodes along the tract profile, between the ROIs. We focus on the central portion of the tract delimited by the ROIs because this is where diffusion data is most reliable and less variable between participants (Yeatman et al. 2012b). At each node, FA was calculated by a weighted average, such that streamlines traveling at the core of the tract are weighted more heavily than streamlines further from the core, as they are more likely to be a member of the tract.

We then used the FA values of each of the tracts of interest to evaluate the extent to which each of the ten tracts can predict spelling performance. As a first step, we evaluated the contribution of mean tract-FA to spelling accuracy scores using a forward and backward stepwise linear regression model. For each participant and tract, FA values in the 100 nodes along the tract were averaged (tract-FA) and defined as predictor variables of spelling accuracy scores. The regression was run using the MATLAB function ‘stepwiselm’, with thresholds to add or remove variables set to *p* < 0.05 (*p*-Enter) and *p* > 0.1 (*p*-Remove) at each step.

#### Association between spelling and tract lateralization

To evaluate whether spelling performance was associated with the degree of tract lateralization, we calculated Spearman’s correlations between spelling scores and the LI of each tract. This analysis was calculated for the full sample (*N* = 73). The FDR was controlled at 5% across the five tracts.

### Spelling and white matter analyses in high- and low- performing spellers

Because the distribution of spelling accuracy scores was not normal across individuals (as indicated by a Shapiro–Wilk test) but rather looked bimodal, we fit a Gaussian mixture model (GMM) to the spelling accuracy data, to test whether a two-component GMM better describes this distribution than a single Gaussian. Model fitting was performed using the MATLAB function ‘fitgmdist’ from the Statistics and Machine Learning Toolbox. To determine which model fit the data better, we compared their Akaike information criteria (AIC). This analysis indicated that the distribution was better fit by a bimodal model rather than by a single Gaussian (see “Results”, "Spelling accuracy scores"). We therefore followed up our analyses by examining spelling effects in two subsamples, dividing the whole sample into two groups based on spelling accuracy scores – high- and low-performing speller groups. Specifically, the criterion for the division into two groups was the spelling score that gave rise to the local minimum of the bimodal model (see Fig. 2b). To compare performance on additional cognitive measures between these two groups, we used a set of two-tailed, two-sample t-tests, controlling the FDR at 5% across seven comparisons. Further analyses described below were conducted between and within these groups.

#### Spelling error analysis

To examine whether the two groups showed different patterns of spelling error types, we analyzed the phonological and orthographic distances (described in "Orthographic and phonological distance") using a linear mixed effects analysis, performed by the MATLAB function ‘fitlme’. Distance values of all responses were defined as the predicted variable, excluding outlier participants who deviated more than 2.5 standard deviations from the mean distance value of each group and error type. As fixed effects, we entered Group, Error Type (phonological, orthographic), and the Group × Error Type interaction. As random effects, we had intercepts per subject and per item. The *p*-value was obtained by the likelihood ratio test of the full model with the interaction effect against the model without the interaction effect.

#### Along-tract spelling associations

Next, we tested whether the groups showed different white matter associations with spelling. For each group separately, we estimated the correlation between spelling accuracy scores and FA along the tracts that predicted spelling scores best (as indicated by the regression model described in Sect. "Spelling-white matter association analyses"). Spearman's correlation coefficients were calculated between spelling accuracy and FA in each node of the tract profile. To account for multiple comparisons (100 nodes along each tract), we applied a nonparametric permutation method for significance correction, resulting in a family-wise error (FWE) corrected alpha level of 0.05 (Nichols and Holmes 2002). In accordance with this method, a significant correlation was detected only if there was a sufficiently large cluster of consecutive nodes, each showing a correlation with *p*_*uncorrected*_ < 0.05. The required cluster size is calculated by a nonparametric permutation algorithm, yielding a FWE corrected alpha level of 0.05. We further corrected for the four comparisons introduced by the two tracts and the two groups analyzed. To this end, the FDR was controlled at 5%, calculated over four *p*-values: In tracts that contained a significant cluster, the *p*-values were those of the correlation between spelling scores and FA values averaged across the nodes that constitute a significant cluster (cluster-FA). If there were no significant clusters within the tract, the *p*-values were those of the correlation between spelling scores and tract-FA.

To evaluate the specificity of significant white matter associations with spelling performance, we assessed the contribution of additional cognitive abilities to the correlations with FA, including lexical-semantic knowledge (evaluated by the vocabulary test), phonological awareness (evaluated by the spoonerisms task), and reading (evaluated by the TOWRE SWE component). To this end, significant correlations with FA were followed up using a multiple regression model that included spelling, vocabulary, spoonerisms, and TOWRE SWE task scores as predictor variables of cluster-FA. To verify that these models did not contain multi-collinearity, we calculated the variance inflation factors (VIF) for each of the variables (Johnston et al. 2018). Regression analyses were carried out using the MATLAB function ‘fitlm’ and VIFs were calculated using JASP (JASP Team 2024).

Additionally, we examined whether the two groups showed microstructural differences in these tracts. For each tract, we compared the average FA profile of each group, using a series of two-tailed, independent sample t-tests, calculated at each node along the tract (FWE corrected, *p* < 0.05).

#### Tract lateralization

To examine whether the degree of tract asymmetry differed between the groups, we compared the LIs of each tract between the groups using a two-tailed, two-sample t-test. We controlled the FDR at 5% across the five tracts.

## Results

### Spelling accuracy scores

**Fig. 1:**
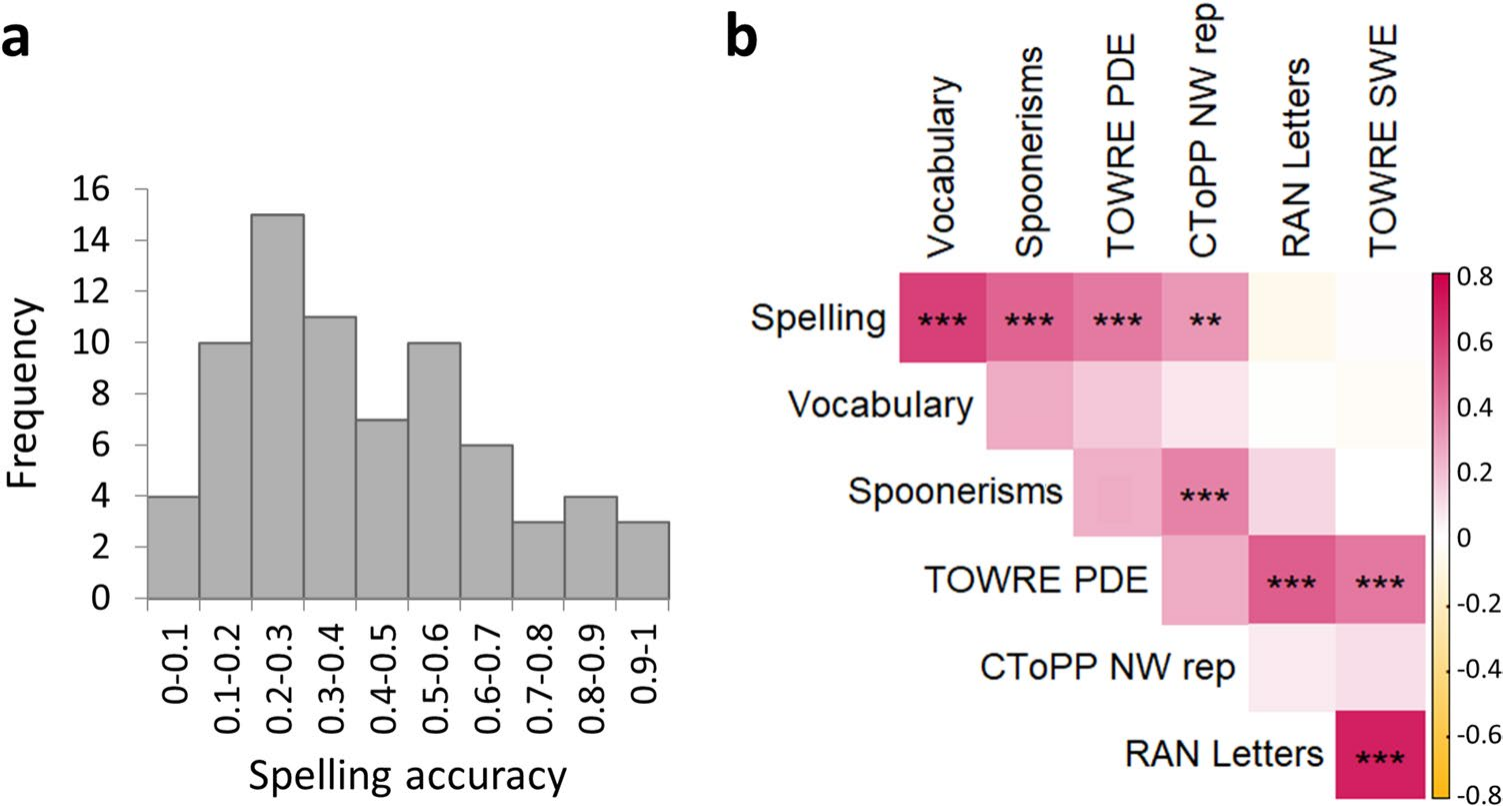
Behavioral results in the full sample (*N* = 73). **a** Distribution of spelling accuracy scores. The x-axis denotes the proportion of correctly spelled items out of 40 trials. **b** Heatmap depicting Spearman's correlation coefficients between pairs of behavioral measures (see “Materials and methods”, “Additional cognitive measures” for detailed task descriptions). The top row indicates that spelling scores significantly correlated with vocabulary and phonologically reliant tasks. RAN scores were multiplied by − 1 for consistency with the direction of all other cognitive measures. **p < .01, ***p < .001, FDR-controlled (q < 0.05). *TOWRE* Test of Word Reading Efficiency, *SWE* sight word efficiency, *PDE* phonemic decoding efficiency, *CToPP* Comprehensive Test of Phonological Processing, *NW rep* nonword repetition, *RAN* Rapid Automatized Naming

Spelling accuracy scores (on a scale of 0 to 1) were widely distributed across individuals, ranging from 0.075 to 0.975 (Fig. 1a; mean accuracy = 0.41, *SD* = 0.23; see Table S2 for ranges, means, and *SD*s on all cognitive subtests included in the analysis). There was also considerable variability in spelling accuracy across items (Fig. S2). Individual spelling accuracy scores were significantly correlated with scores on the vocabulary, spoonerisms, pseudoword reading (TOWRE PDE), and nonword repetition tests (Fig. 1b; FDR controlled at a level of q < 0.05). Non-significant correlations were found between spelling accuracy and word reading (TOWRE SWE) and between spelling accuracy and RAN (Fig. 1b).

**Fig. 2:**
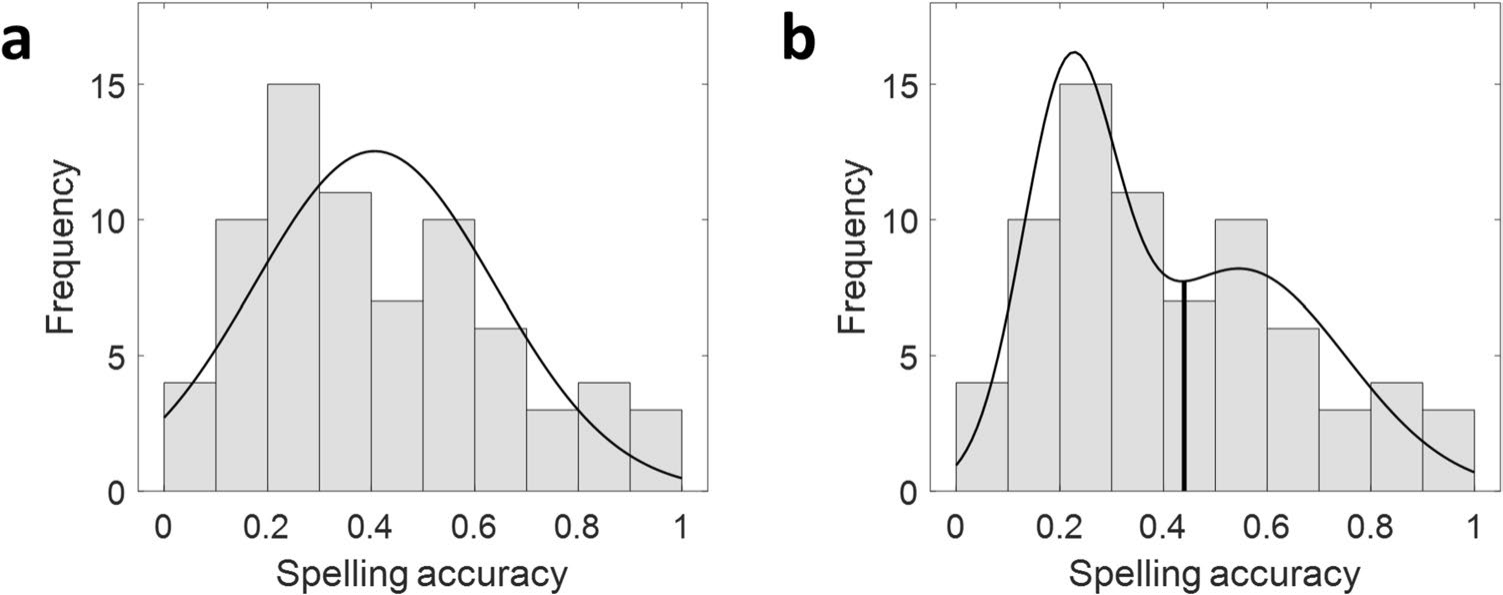
Distribution of spelling accuracy scores is better fit by a two-component GMM rather than one. A distribution composed of two Gaussians (**b**; µ_1_ = 0.22, σ_1_ = 0.008, µ_2_ = 0.55, σ_2_ = 0.041) yielded a better fit to the data, with a considerably lower Akaike information criterion (AIC = − 8.64) than a single Gaussian distribution (**a**; µ = 0.41, σ = 0.054, AIC = -1.87). We used the spelling score that gave rise to a local minimum of the two-component GMM (marked with a vertical black line in **b**) as the criterion to divide participants into two groups, low- and high-performing spellers, for further analyses (shown in Figs. 5 and 6). *GMM* Gaussian mixture model

A closer examination of Fig. 1a suggested that the distribution of spelling scores deviated considerably from the Gaussian distribution. Indeed, a Shapiro–Wilk normality test confirmed that the distribution was not normal (W = 0.94, *p* = 0.003, significantly rejecting the hypothesis of a normal distribution). Fitting a Gaussian mixture model (GMM) to the data revealed that a two-component GMM better fits the distribution than a single Gaussian distribution, with a considerably lower Akaike information criterion (AIC = − 8.64, against AIC = -1.87; see Fig. 2a-b).

With this in mind, we first analyzed the full sample, then followed up with group analyses. Specifically, the most relevant pathways were identified using a stepwise regression analysis performed over the full sample (see “Materials and methods”, "Spelling-white matter association analyses"). These pathways were later inspected within each of two groups: low-performing spellers (*n* = 41) and high-performing spellers (*n* = 32). The criterion to assign participants to the groups was the spelling score that gave rise to a local minimum of the two-component GMM (criterion = 0.44, see Fig. 2b). Notably, these two groups did not show different word reading abilities as measured by the TOWRE SWE (see Table S3 for a comparison of other cognitive measures between these groups).

### White matter associations with spelling

**Fig. 3:**
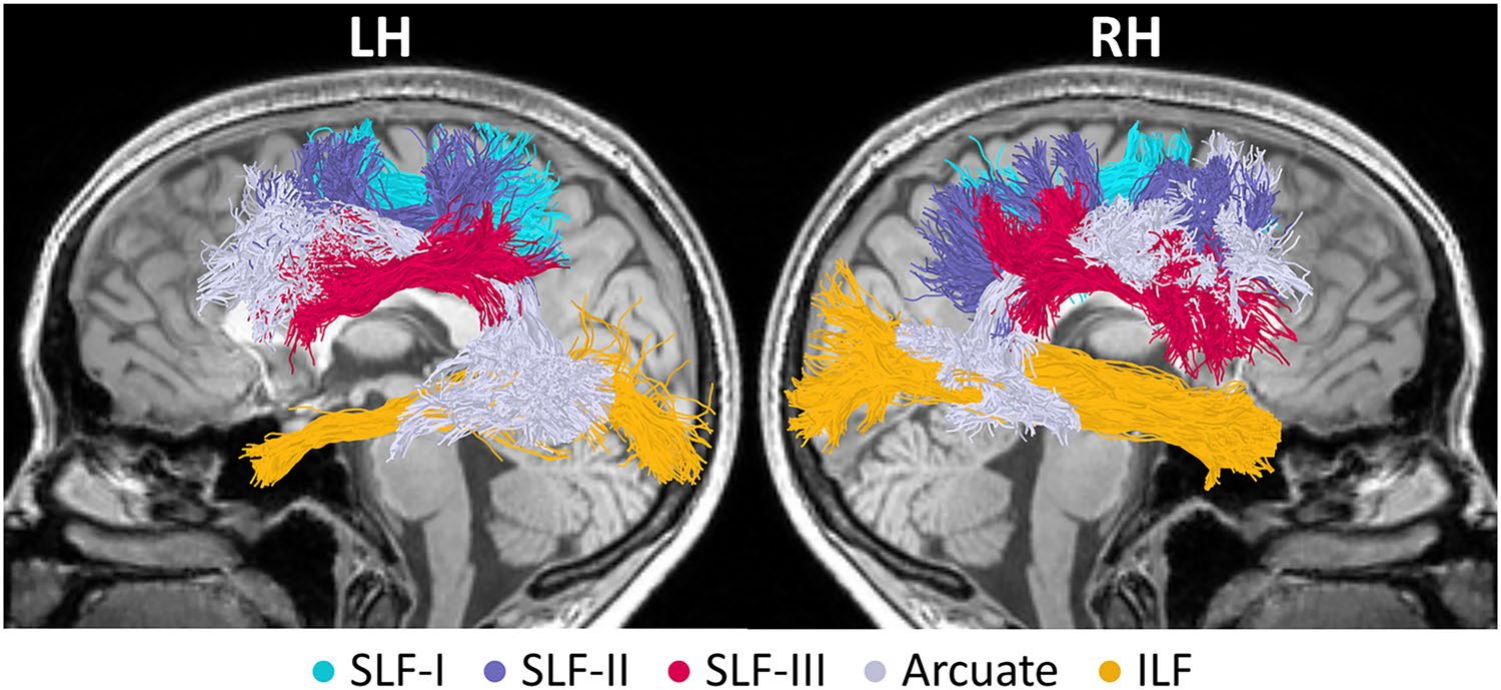
Tracts of interest. Tracts were identified using CSD modeling coupled with probabilistic tractography in each participant’s native space. All tracts are shown bilaterally for a single participant (female, 21), overlaid on a T1 midsagittal image. Dorsally, we segmented the three branches of the superior longitudinal fasciculus (*SLF-I* in cyan, *SLF-II* in purple, *SLF-III* in magenta) and the frontotemporal arcuate fasciculus (light gray). Ventrally, we segmented the inferior longitudinal fasciculus (*ILF,* yellow). *LH* left hemisphere, *RH* right hemisphere, *CSD* constrained spherical deconvolution

**Fig. 4:**
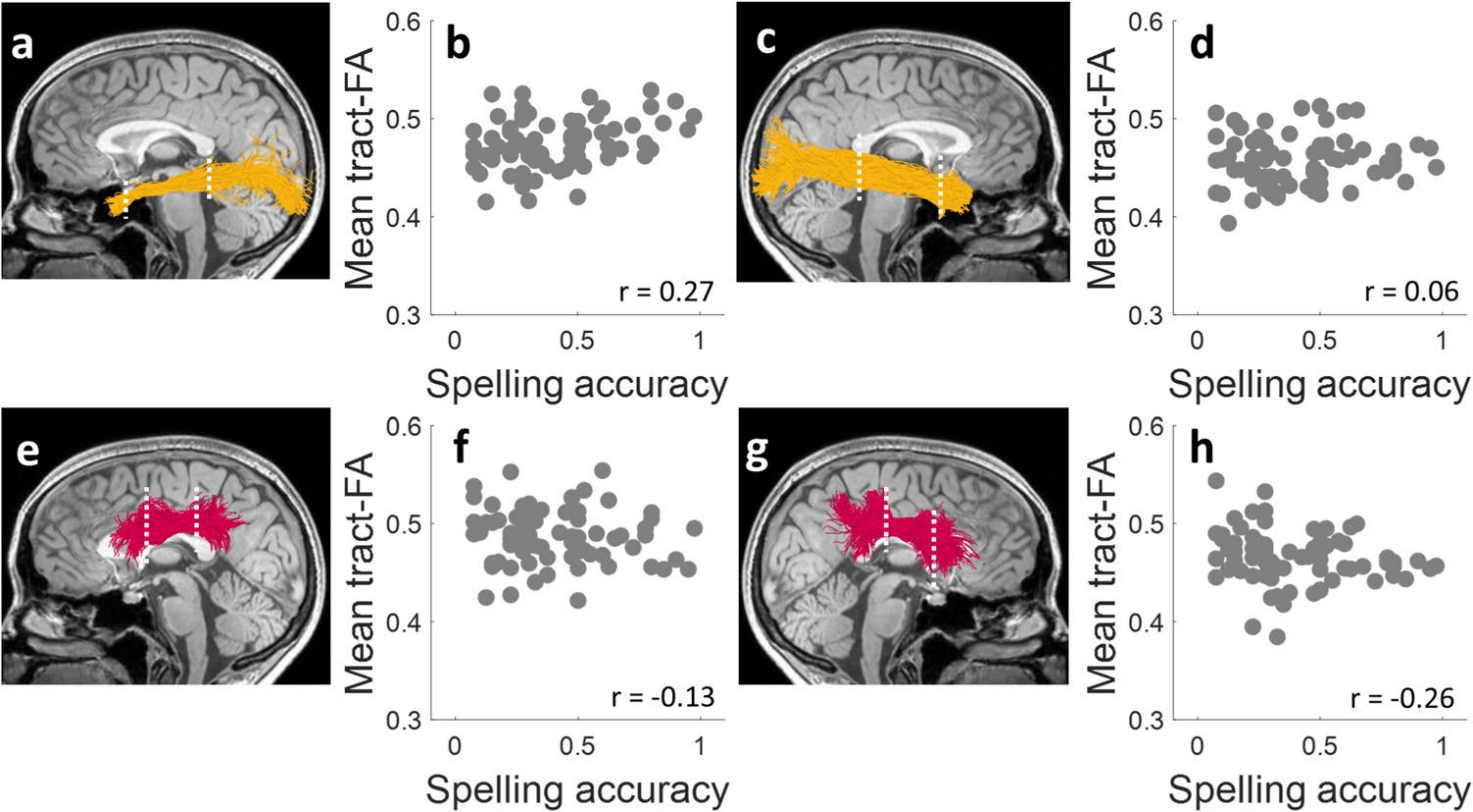
Spelling accuracy is predicted by tract-FA in the left ILF (**a**, **b**) and right SLF-III (**g**, **h**). Tractograms depict the tracts in a single participant (female, 21), with white dashed lines marking ROIs. Tract-FA was calculated as the average of FA values between the ROIs. Scatter plots show the associations between spelling accuracy scores and tract-FA (*N* = 73, except for the left ILF where *n* = 72). In a stepwise linear regression model including 10 tracts of interest, tract-FA values of the left ILF (**a**, **b**) and the right SLF-III (**g**, **h**) were identified as predictive of spelling accuracy scores across the full sample (see “Materials and methods”, "Spelling-white matter association analyses" for model calculation and Table S4 for model output). Also presented are the homologous tracts: right ILF (**c**, d) and left SLF-III (**e**, **f**), which did not predict spelling scores. r values are Spearman’s correlation coefficients. A similar visualization for the bilateral SLF-I, -II, and arcuate fasciculus is provided in Fig. S3

Nearly all tracts were successfully identified in all 73 participants (see Fig. 3 for visualization in a single participant). The only exception was the left ILF, which was identified in 72 out of 73 participants. For this reason, one participant was excluded from any analysis that included the left ILF. Quality assurance checks confirmed that all identified tracts were segmented correctly; therefore, all identified tracts were included in the analyses. To assess which of the ten tracts of interest predicted spelling accuracy in the full sample, tract-FA data from all participants and tracts were entered into a stepwise linear regression model, with spelling accuracy as the dependent variable (*n* = 72 participants, 10 tracts). This model identified tract-FA of the left ILF and right SLF-III as predictive of spelling accuracy scores, suggesting that these two pathways are relevant to spelling performance (see Table S4). Figure 4 visualizes the association patterns between spelling accuracy and tract-FA within these tracts and their homologs (similar information is provided for the remaining tracts in Fig. S3).

**Fig. 5:**
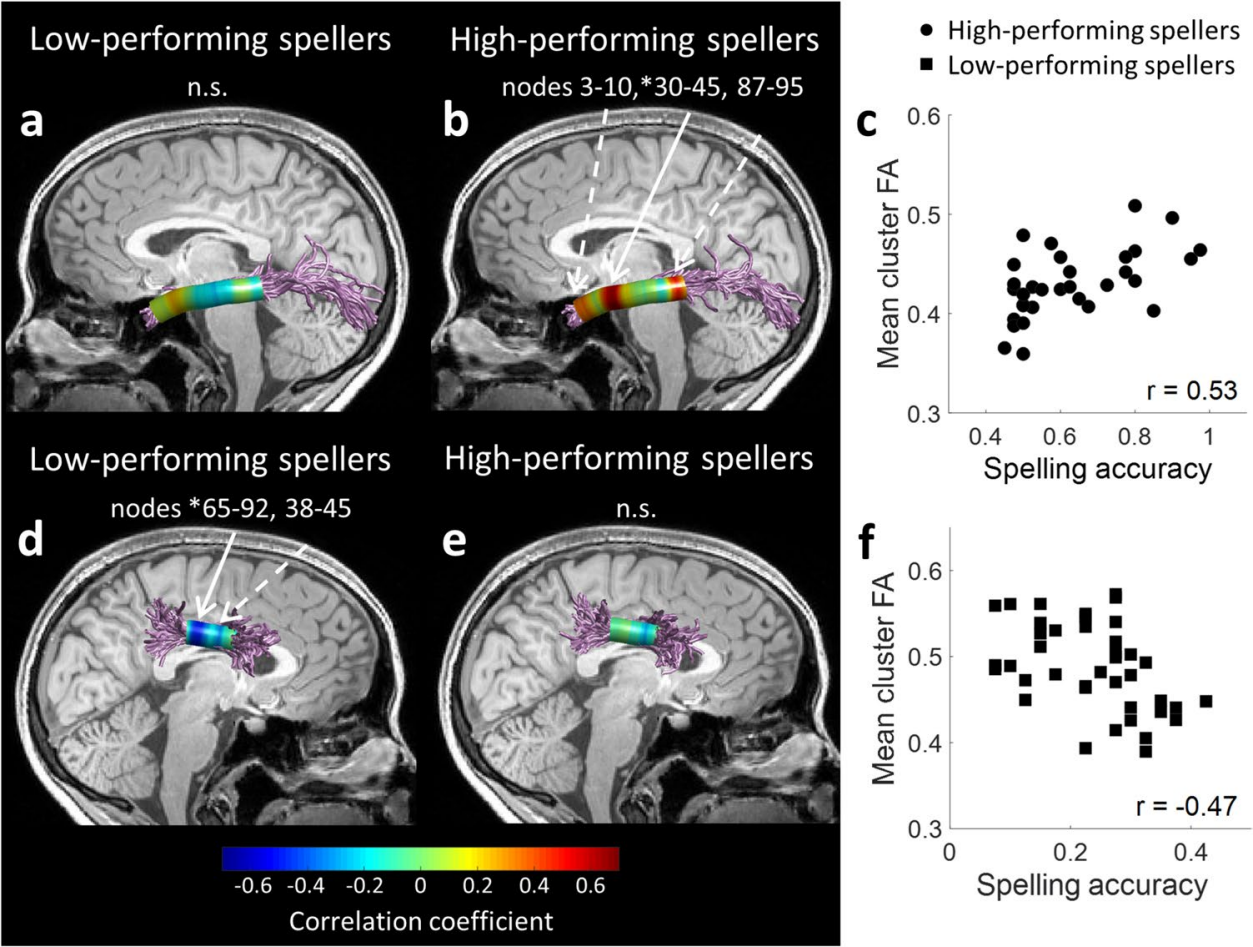
Distinct white matter association patterns among high- and low- performing spellers. Spearman's correlation coefficients are visualized between spelling scores and FA for 100 nodes along the left ILF (**a**, **b**) and right SLF-III (**d**, **e**) among high-performing spellers (*n* = 32; **b**, **e**) and low-performing spellers (*n* = 41; **a**, **d**). Solid arrows denote the location of significant clusters after family-wise error correction across the 100 nodes (node numbers of significant clusters are marked by *). Dashed arrows indicate nodes with *p* < 0.05, uncorrected. Scatter plots show the associations between spelling scores and significant cluster-FA in the left ILF among high-performing spellers (**c**) and in the right SLF-III among low-performing spellers (**f**). r values are Spearman’s correlation coefficients. *n.s.* no significant clusters in the left ILF among low-performing spellers (**a**) and in the right SLF-III among high-performing spellers (**e**)

We further explored the spelling associations within the left ILF and right SLF-III by separating the sample into two groups, based on the bimodal distribution of spelling scores described above. Indeed, an analysis of the correlations between spelling performance and FA for the nodes along the tracts revealed distinct association patterns in each group. Within the left ILF, spelling scores were positively correlated with FA values only in high-performing spellers, while low-performing spellers did not show any significant cluster of nodes in this tract. Conversely, within the right SLF-III, spelling scores were negatively correlated with FA values only among low-performing spellers, while no significant clusters of nodes were found in high-performing spellers. See Fig. 5 for a visualization of the locations of significant clusters (panels b, d) and the distribution of individual values, including the correlation coefficients of significant clusters (panels c, f). All significant findings reported here are significant at *p* < 0.01, FWE corrected across 100 comparisons, controlling the FDR at q < 0.05 across the two tracts and two groups (see “Materials and methods”, "Along-tract spelling associations").

To examine the specificity of the observed white matter associations with spelling performance compared to other related cognitive measures, significant correlations were followed up with multiple regression models. Each model predicted the mean FA of a significant cluster (dependent variable) based on the following predictor variables: spelling, lexical-semantic knowledge (vocabulary), phonological awareness (spoonerisms), and word reading (TOWRE SWE). VIF scores of all variables within each model were < 2, indicating that the models did not contain excessive collinearity (Johnston et al. 2018). As reported in Table 1, none of these additional variables made a significant contribution to predicting cluster-FA in either model (*p* > 0.05). This indicates that the correlations were driven primarily by spelling abilities.

**Table 1:** Specificity of white matter associations with spelling

Predictor	B^1^	SE	β^2^	*t*	*p*	VIF
(A) Left ILF, High-performing spellers (*n* = 32)						
(Constant)	0.35	0.09		3.98	<0.001	
Spelling	0.11	0.05	0.52	2.36	0.026	1.920
Vocabulary	0.03	0.08	0.07	0.40	0.693	1.133
Spoonerisms	− 0.001	0.06	− 0.004	− 0.02	0.985	1.949
TOWRE SWE	− 0.0002	0.0004	− 0.08	− 0.45	0.655	1.119
Overall regression: R^2^ = 0.311, *F*(4, 36) = 3.05, *p* = 0.0339						
(B) Right SLF-III, Low-performing spellers (*n* = 41)						
(Constant)	0.58	0.09		6.27	<0.001	
Spelling	− 0.27	0.08	− 0.51	− 3.42	0.002	1.138
Vocabulary	− 0.01	0.09	− 0.02	− 0.13	0.899	1.048
Spoonerisms	0.08	0.04	0.29	1.95	0.059	1.144
TOWRE SWE	− 0.0008	0.0005	− 0.20	− 1.40	0.169	1.051
Overall regression: R^2^ = 0.286, *F*(4, 26) = 3.6, *p* = 0.0143						

A multiple regression model predicting cluster-FA in left ILF in high-performing spellers (A) and right SLF-III in low-performing spellers (B) from spelling, vocabulary, spoonerisms, and TOWRE sight word efficiency (SWE). The contribution of all predictor variables except for spelling accuracy was non-significant in both cases (*p* > 0.05)

^1^Unstandardized Coefficient, ^2^ Standardized Coefficient, *VIF* variance inflation factor

For completeness, we compared the along-tract FA profiles of each group, examining whether there are microstructural differences between the groups (Fig. S4). The group profiles overlapped nicely, showing no statistically significant group difference between the anisotropy values along the tracts. This demonstrates that the differences between the groups in the correlation between the anisotropy values and the spelling scores are not due to differences in the anisotropy values themselves.

### Spelling error analysis

**Fig. 6:**
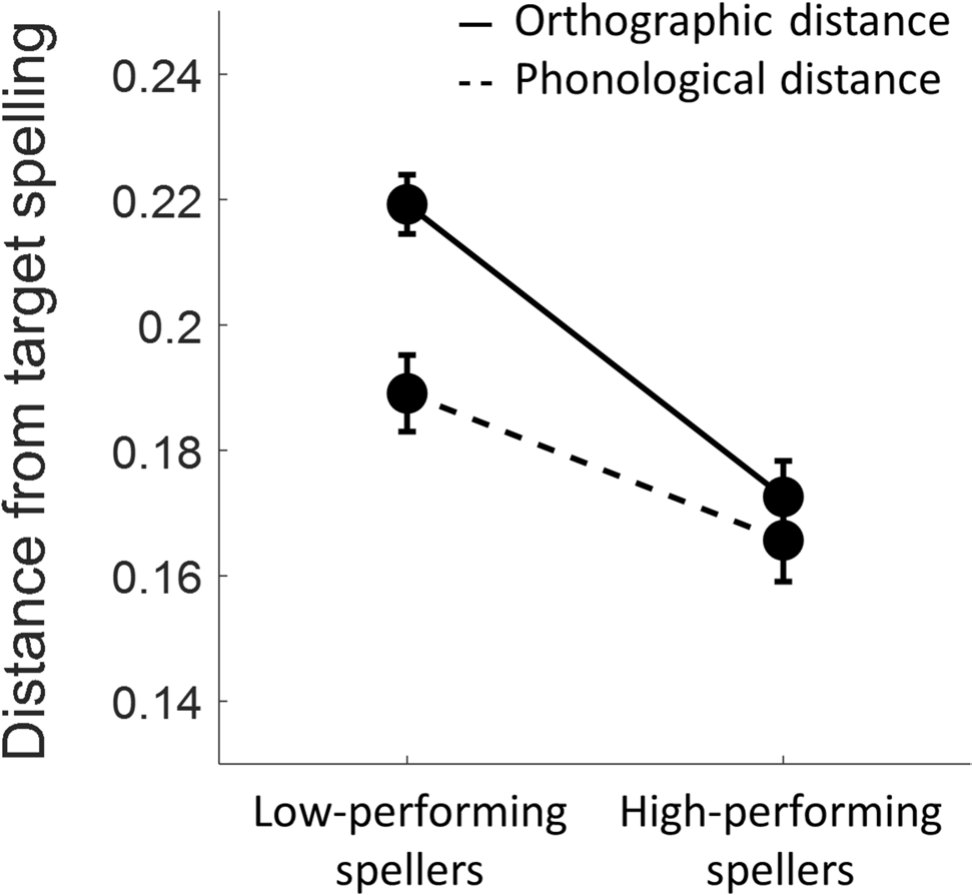
Group differences in patterns of spelling errors. Mean orthographic and phonological distances of spelling errors are presented for each group (see “Materials and methods”, "Orthographic and phonological distance", for the calculation of orthographic and phonological distances). Error bars denote ± 1 standard error of the mean. A linear mixed effects model revealed a significant interaction effect between Group and Error type, demonstrating a larger divergence between the groups in orthographic distance compared to phonological distance (*p* < 0.01; see “Materials and methods”, "Spelling error analysis" for LME model calculation)

To better characterize participants’ performance in terms of the specific cognitive processes involved in spelling, we quantified the orthographic distance and the phonological distance of each response from the target spelling. The mean orthographic and phonological distances of the errors in each group are presented in Fig. 6 (excluding three outliers, see “Materials and methods”, "Orthographic and phonological distance"). The individual data are provided in Fig. S5. Using a linear mixed effects model, we found a significant interaction between Group and Error type, such that the group difference in orthographic distance was larger than the group difference in phonological distance (χ^2^(1) = 8.1, *p* = 0.00044). Simple effects of Group were found significant (*p* < 0.001, Bonferroni corrected for two independent samples t-tests) both in orthographic distance and phonological distance (*t*(68) = 12.9, *p* < 0.0001; *t*(68) = 10.7, *p* < 0.0001, respectively), but the significant interaction indicates that the difference in orthographic distance was significantly larger. These results indicate that the groups diverged more extensively in the integrity of their orthographic-lexical representations, compared to the phonological plausibility of their responses.

### Tract lateralization

LIs of all tracts are presented in Fig. S6a. Both the SLF-III and the ILF showed significant right lateralization in terms of number of streamlines (SLF-III: mean LI = 0.24, *SD* = 0.14, *t*(72) = − 14.5, *p* = 3.9 × 10^–23^; ILF: mean LI = 0.46, *SD* = 0.22, *t*(71) = − 17.3, *p* = 3.4 × 10^–27^). The arcuate and the SLF-I showed significant left lateralization, and the SLF-II did not show any significant lateralization (arcuate: mean LI = -0.27, *SD* = 0.18, *t*(72) = 12.8, *p* = 3.4 × 10^–20^; SLF-I: mean LI = -0.19, *SD* = 0.37, *t*(72) = 4.5, *p* = 2.5 × 10^–5^; SLF-II: mean LI = 0.03, *SD* = 0.18, *t*(72) = − 1.2, *p* = 0.25). All significant effects passed the Bonferroni-adjusted alpha level (*p* < 0.01). These patterns of tract lateralization are largely consistent with published reports (Amemiya et al. 2021; Thiebaut de Schotten, ffytche, et al. 2011).

Next, we evaluated whether the degree of lateralization of each pathway was predictive of spelling accuracy scores. We found that the lateralization of the SLF-III was moderately correlated with spelling scores (r = 0.25, *p* = 0.03), but none of the LI-correlations was significant after correction for the five tracts. We further assessed whether low- and high-performing spellers showed different lateralization patterns. In a group comparison of LI values, high-performing spellers showed a slightly stronger right lateralization of the SLF-III compared to low-performing spellers (mean LI_high_ = 0.28, *SD* = 0.12; mean LI_low_ = 0.20, *SD* = 0.14; *t*(71) = 2.4, *p* = 0.018). However, this result, too, did not survive correction for five comparisons. Individual values and the pattern of covariation between spelling accuracy and LI values in each tract are visualized in Fig. S6b-d.

In sum, we found that, in terms of number of streamlines, both spelling-associated tracts (SLF-III and ILF) were right-lateralized. The degree of lateralization of the SLF-III correlated with spelling accuracy scores and differed between groups of high- and low-performing spellers, suggesting that the hemispheric balance of the SLF-III might play a role in spelling. However, the latter two results did not survive multiple comparison corrections.

## Discussion

The current study examined the white matter pathways associated with spelling abilities in neurotypical English-speaking adults. Within the analyzed pathways, the anisotropy of one ventral tract and one dorsal tract was predictive of spelling performance on a difficult spelling-to-dictation task. Observing that spelling scores were distributed bimodally across individuals, a follow-up analysis revealed distinct association patterns in high- and low- performing spellers. The ventral tract association, identified in the left ILF, was significant only in high-performing spellers, while the dorsal tract association in the right SLF-III was significant only in low-performing spellers. These groups also differed in the pattern of their error types, diverging more extensively in terms of the lexical-orthographic distance of their responses from the correct spelling, compared to the phonological plausibility of their responses.

To date, only a handful of studies have investigated the white matter pathways that support spelling, while numerous dMRI studies have investigated reading (for reviews see, e.g., Ben-Shachar et al. 2007; Caffarra et al. 2021; Vandermosten et al. 2012b; Wandell and Le 2017; Wandell and Yeatman 2013). Importantly, despite some shared neural substrates, spelling involves different cognitive components than written word recognition. In the current sample, spelling scores were not significantly correlated with word reading, demonstrating the distinction between these abilities in healthy adults. Our findings are generally in line with the few published reports on spelling-associated white matter tracts, which have focused primarily on atypical populations. Ventrally, we found an association with the left ILF, which was previously associated with spelling abilities in children with dyslexia and in skilled neurotypical readers (Banfi et al. 2019; Cheema et al. 2022). This pathway provides connectivity between anterior temporal and occipitotemporal regions, specifically the VWFA, which is consistently activated during spelling tasks (DeMarco et al. 2017; Ludersdorfer et al. 2015; Planton et al. 2013; Purcell et al. 2011b; Rapp and Dufor 2011; Rapp and Lipka 2011). Dorsally, we found an association with the right SLF-III, consistent with previous studies reporting spelling associations with the right SLF in children with dyslexia and dysgraphia (Banfi et al. 2019; Gebauer et al. 2012). The SLF connects parietal and frontal regions which constitute a major part of the spelling cortical network as identified in functional imaging studies, lesion studies and cortical stimulation studies (Baldo et al. 2018; Planton et al. 2013; Purcell et al. 2011b; Rapp et al. 2016; van Ierschot et al. 2018).

### Distinct neurocognitive processes in spelling

The distinct patterns of white matter associations detected in high- and low- performing spellers suggest that these individuals may rely on different neurocognitive processes when performing a challenging spelling task. Most cognitive models of written word production describe two routes to spell a word: lexical and sublexical (Baxter and Warrington 1985; Beauvois and Dérouesené, 1981; Goodman-Schulman and Caramazza 1987; Shallice 1981). The fact that high- and low- performing spellers diverged primarily in the lexical-orthographic distance between their errors and the correct spelling, suggests that high-performing spellers had more accurate, stably stored, lexical-orthographic representations of the words, allowing them to rely on the lexical spelling route, which is the efficient way to spell known words. Low-performing spellers presumably lacked stable lexical-orthographic representations of these low-frequency words, and therefore could not rely on lexical processes, but instead may have made use of the sublexical spelling route. Because this test incorporates a large proportion of orthographically irregular words with low probability mappings between phonological and orthographic forms, relying on phoneme-to-grapheme conversion rules to spell these words is likely to result in phonologically plausible but incorrect spellings.

In high-performing spellers, spelling accuracy correlated with FA in the left ILF. Previous white matter studies report associations of the ILF with lexical-semantic aspects of language processing (e.g., Cummine et al. 2015; Nugiel et al. 2016), as well as with morphological decomposition (Yablonski et al. 2019, 2021; Yablonski and Ben-Shachar 2020). The ILF connects anterior temporal areas with occipitotemporal areas, including the VWFA (Epelbaum et al. 2008; Wandell et al. 2012; Wandell and Yeatman 2013), a region that was argued to host lexical-orthographic representations (Cohen and Dehaene 2004; Glezer et al. 2009; Purcell et al. 2014; Rapp et al. 2016; Rapp and Lipka 2011; Thesen et al. 2012; Tsapkini and Rapp 2010). The VWFA is activated when performing both reading and spelling tasks (Dębska et al. 2019; Purcell et al. 2017; Purcell et al. 2011a; Rapp and Dufor 2011; Rapp and Lipka 2011). In spelling, it is linked to central (rather than peripheral) spelling processes (Ludersdorfer et al. 2015; Planton et al. 2013; Purcell et al. 2011a, b). The ILF belongs to the ventral language stream, which is thought to map sensory or phonological representations onto lexical-conceptual representations, according to the dual-stream model of the functional anatomy of language (Hickok and Poeppel 2007). In the context of spelling, such mapping would be part of spelling via the lexical route. Importantly, in our data, the correlation in the left ILF was not explained by other cognitive measures, including vocabulary, word reading, or phonological awareness. Taken together, the association found in the left ILF among high-performing spellers suggests that this pathway is involved in lexical spelling processes.

Conversely, in low-performing spellers, spelling scores correlated with FA in the right SLF-III. The SLF is a frontoparietal pathway, which is part of the dorsal language stream, thought to compute phonological-level processing and to map phonological representations onto motor representations for production (Hickok and Poeppel 2007). It is possible that the association with the right SLF-III among low-performing spellers reflects their reliance on phonological (sublexical) processes in the spelling task. Consistent with this interpretation, sublexical spelling processes have been previously associated with activation in bilateral frontal and parietal cortical areas (DeMarco et al. 2017). In our data, this correlation, too, was not explained by vocabulary, word reading, or phonological awareness. These results, together with the error analysis, which implies that low-performing spellers have relatively poor lexical spelling knowledge and therefore may rely more heavily on the sublexical spelling route, suggest that the right SLF-III is involved in sublexical spelling processes, particularly in low-performing spellers.

### Right hemisphere associations with spelling

Our results emphasize the involvement of the right hemispheric frontoparietal connections in spelling, consistent with previous white matter studies (Banfi et al. 2019; Gebauer et al. 2012). This is intriguing, given that the dorsal stream, and specifically spelling-associated dorsal cortical regions, are mostly left dominant. We therefore discuss possible accounts of the involvement of the right SLF-III in spelling. In our data, the right-hemisphere spelling association was significant only in low-performing spellers. Altered properties of the right SLF and other right-hemispheric homologs of language-related tracts were previously shown in children and adults with spelling and reading difficulties, but the direction of these differences is inconsistent (Banfi et al. 2019; Gebauer et al. 2012; Hoeft et al. 2011; Steinbrink et al. 2008). For example, dyslexic children showed higher FA in the right SLF compared to controls (Banfi et al. 2019). Additionally, long-term reading improvement in children with dyslexia was predicted by FA in the right SLF and arcuate fasciculus, while typical readers did not show this pattern (Hoeft et al. 2011). In another study, spelling-impaired children showed lower FA in right-hemispheric regions, including the right SLF, compared to controls (Gebauer et al. 2012). Finally, spelling intervention in these spelling-impaired children resulted in a right-hemispheric FA increase (Gebauer et al. 2012).

The involvement of the right hemisphere in language processing has been traditionally described as a compensatory mechanism for the decreased involvement of the left-hemispheric regions in impaired populations (Hamilton et al. 2011). While this discussion has been focused primarily on functional activation patterns, compensatory processes may well involve long-term plasticity that affects the microstructural properties of white matter pathways as well (Zatorre et al. 2012). Accordingly, the negative correlation we identified in the right SLF-III of low-performing spellers could be viewed as part of a maladaptive mechanism that is ineffective in compensating for the weaker spelling abilities of this group (see also "Interpreting correlation directions in diffusion MRI data" below). Contrary to the prior findings reviewed above, our participants reported no history of reading or language difficulties. Our participants diverged primarily on their spelling scores, and not so much on their word reading scores. In fact, no significant difference was found between the low and high spelling groups on word reading, and word reading did not explain the correlation between spelling scores and FA in the right SLF-III. Therefore, the findings we report in the right hemisphere of low-performing spellers seem to reflect a non-successful attempt to compensate for their spelling abilities, not their reading abilities. Specifically, low-performing spellers may show overreliance on phoneme-to-grapheme conversion mechanisms in the face of weak lexical mechanisms, despite the fact that the phoneme-to-grapheme mechanisms cannot yield lexically correct responses for words with unpredictable spellings. This may be due to inherent weakness in left hemispheric lexical mechanisms, or to an over-developed sublexical system.

Another possibility for the right frontoparietal association detected in low-performing spellers could be that it reflects the involvement of a domain-general network. Such a “multiple-demand” system comprised of bilateral frontal and parietal cortical areas was shown to be involved in executive control and selective attention (Duncan 2010, 2013; Fedorenko et al. 2013). It is specifically recruited as the difficulty of the task increases, as shown within various language and non-language tasks. Perhaps increased effort in low-performing spellers requires persistent recruitment of such a domain-general network.

Finally, we also observed a trend (non-significant when correcting for multiple comparisons) of an association between spelling scores and the degree of SLF-III lateralization, in terms of number of streamlines. Further, the degree of SLF-III lateralization differed between high- and low-performing spellers. Other pathways, including other branches of the SLF, did not show such an association between the extent of lateralization and spelling performance. These results suggest that the hemispheric balance of the SLF-III, not only the properties of the right SLF-III per-se, may be important for certain spelling processes. This idea remains to be tested in future studies as the size of the effect here does not warrant stronger conclusions.

### Segmenting the three branches of the SLF

The current study examined the distinct association of each of the three SLF branches with spelling abilities. We found spelling association in the SLF-III, the ventral branch of the SLF complex, while no significant associations were displayed in the SLF-II or -I. Traditionally, the SLF was considered a single major frontoparietal pathway. To date, most dMRI studies have segmented the SLF as one complex, linked to various cognitive functions such as arithmetic abilities (Tsang et al. 2009; Van Beek et al. 2014), visuospatial attention (Thiebaut de Schotten et al. 2011b), and working memory (Karlsgodt et al. 2008; Van Beek et al. 2014). Consistent with primate studies, anatomical and dMRI studies in humans support the division of the SLF into three branches, which are assumed to vary in their functional involvement in neurocognitive processes (Amemiya et al. 2021; Makris et al. 2005; Petrides and Pandya 1984; Schmahmann and Pandya 2007; Schurr et al. 2020; Thiebaut de Schotten et al. 2012). We expected that the SLF-III or -II are more likely to convey spelling information than the SLF-I, as they connect cortical regions better known for their contribution to language functions. Specifically, the SLF-II connects dorsolateral frontal regions with caudal-inferior parietal regions, and the SLF-III connects prefrontal and premotor areas (BA44/9, including pars opercularis, inferior frontal junction, and ventral precentral gyrus) with the supramarginal gyrus and angular gyrus. Based on these connected regions, the SLF-III in particular was long hypothesized to play a role in language production (Makris et al. 2005).

The fibers of the SLF-III run contiguously with the fibers of the arcuate fasciculus (anterior and long segments, Catani et al. 2005), hugging the Sylvian fissure and insula, and terminating in overlapping frontal regions. The frontotemporal arcuate fasciculus was included in our analysis as it connects frontal and temporal regions within the spelling-associated cortical network. The arcuate has been associated with language production since the early days of cognitive neuropsychology (Geschwind 1970; Lichtheim 1885; Wernicke 1874) and was linked to phonological awareness and reading skills in dMRI studies (Dick and Tremblay 2012; López-Barroso et al. 2013; Thiebaut De Schotten et al. 2014; Vandermosten et al. 2012a; Yeatman et al. 2012a). We did not find significant associations with spelling performance in the arcuate fasciculus, although it has been linked to spelling in a previous study of spelling-impaired children (Banfi et al. 2019). The current results point to the SLF-III as being more specifically involved in spelling than the arcuate. However, further dMRI studies segmenting the SLF into its separate branches, bilaterally, are required to establish more clearly the involvement of the dorsal white matter pathways in spelling.

### Interpreting correlation directions in diffusion MRI data

In the current study, spelling scores were positively correlated with FA in the left ILF and negatively correlated with FA in the right SLF-III. In high-performing spellers, higher FA in the left ILF was associated with better spelling performance. This finding can be intuitively interpreted under a common view that FA is an index of structural integrity, where higher FA indicates more efficient connectivity (for criticism see Jones et al. 2013). In low-performing spellers, higher FA in the right SLF-III was associated with lower spelling accuracy. This finding could then be explained as an unsuccessful attempt by the right hemisphere to compensate for weak lexical representations, resulting in inaccurate spelling responses, as proposed earlier. Broadly speaking, negative correlations between FA and cognitive measures are not uncommon in dMRI studies, including those that address reading and spelling-related skills (e.g., Arrington et al. 2017; Dougherty et al. 2007; Frye et al. 2011; Yeatman et al. 2012a, b). The direction of FA correlations may stem from various tissue factors that affect FA values, including axonal density, axonal diameter, directional coherence of the fibers and myelin content (Assaf and Pasternak 2008). The interaction between these factors may be expressed differently in the different pathways, and may have a positive or a negative effect on the efficiency of information transfer (Jones et al. 2013). Therefore, any interpretation of the direction of the correlation between FA and spelling is tentative at best.

### Limitations and suggestions for future studies

This study evaluated spelling using typing, which is the prevalent form of written language production among adults nowadays. Typing also provides a simpler response modality, which probes more directly the central (linguistic) processes involved in written language production. Handwriting, which is a common response modality in neuropsychological tests of spelling, is considered a more complex motor task (Weingarten and Nottbusch 2004). Despite clear differences between these modalities, typing and handwriting are assumed to share most spelling processes. For example, different types of grapheme units (e.g., syllables, morphemes, geminates) were shown to influence the kinematics of production similarly in both typing and handwriting (Weingarten and Nottbusch 2004). Neurally, typing and handwriting were shown to share their associated cortical network with only subtle distinctions, which are negligible in the scale of the major white matter pathways investigated here (Higashiyama et al. 2015; Purcell et al. 2011a). However, typing involves parallel motor programming for multiple letters (Salthouse 1986; Salthouse and Saults 1987; West and Sabban 1982), and is prone to peripheral errors (‘typos’) that occur after central spelling processes have retrieved and assembled the representations of the letters to be produced. Since we had no adequate means to distinguish typos from other error types, such errors were potentially included in the overall spelling accuracy scores. However, typos are likely to affect both the orthographic and phonological distances, so they are unlikely to explain differences in error types. Future studies could assess typing quality and typing speed separately from spelling accuracy, so these factors can be carefully controlled.

The spelling task in the current study was comprised of low-frequency words, in order to elicit a wide range of accuracy scores in healthy college students. Poor familiarity with the tested words among participants could have influenced spelling performance, specifically affecting the balance between lexical and sublexical spelling processes. However, the associations found between white matter pathways and spelling performance were not explained by general lexical-semantic knowledge, as evaluated by the vocabulary task. Nonetheless, in future studies we plan to further assess the familiarity of participants with the words they are required to spell.

Finally, the current study focused on white matter pathways associated with spelling in English. It remains an open question whether the current findings would generalize across languages, as typological differences and orthographic features may affect the balance between different sub-processes involved in spelling. For example, one main typological factor affecting spelling systems is orthographic depth (Katz and Frost 1992). English has a deep (opaque) orthography with unpredictable correspondence between spoken and written representations. As such, it contains numerous irregularly-spelled words that mostly rely on memorization (Fischer et al. 1985). Other European languages, like German or Italian, have transparent orthographies in which the majority of words obey simple phoneme-to-grapheme conversion rules, resulting in more predictable spellings with less reliance on memorization. This factor may affect the balance between lexical and sublexical processes in spelling, such that shallower orthographies may enhance sublexical processes while deeper orthographies may enhance lexical processes. Such differences may in turn affect the involvement of phonologically-related dorsal pathways and lexically-related ventral pathways in spelling.

Spelling systems are also affected by morphological structure, which varies across languages. For example, morphological complexity has been shown to facilitate spelling production in English and in Hebrew, as words are processed as morphemic units, enabling a reduction in processing load (Allen and Badecker 1999; Badecker et al. 1990; Yachini and Friedmann 2008). In English, spelling preserves morphological consistencies at the expense of orthographic-phonological regularities, such that morphological cues are beneficial to accurate spelling (Aronoff et al. 2016; Fischer et al. 1985; Heyer 2021; Levesque et al. 2021; Rastle 2019; Ulicheva et al. 2020). In Dutch, which has sparse morphology, morphological cues were found less beneficial to spelling compared to Hebrew, a Semitic language with a highly synthetic morphology (Gillis and Ravid 2006). In morphologically-rich Semitic languages, like Arabic or Hebrew, phonological information is underspecified in the spelling system and morphological knowledge provides crucial cues for accurate spelling (Bar-On and Kuperman 2019; Levin et al. 2001; Ravid 2001; Schiff et al. 2020). These languages have a non-linear morphological structure, such that the root morpheme is embedded within a template morpheme. Recognizing the root may facilitate access to the correct spelling of a word. Previously, we found that sensitivity to morphological information in both Hebrew and English was associated with ventral white matter pathways (Yablonski et al. 2019; Yablonski and Ben-Shachar 2020). We hypothesize that the extent to which spelling is reliant on morphological cues may therefore affect the associations of spelling performance with dorsal and ventral pathways.

To conclude, the current findings highlight the complexity of the neurocognitive architecture of the spelling process. Specifically, spelling is shown to be associated with both dorsal and ventral white matter pathways. The distinct associations detected in low- and high- performing spellers suggest that they rely on different cognitive processes in spelling, such that high-performing spellers rely more on lexical-orthographic representations mediated by ventral tracts, while low-performing spellers rely more on phoneme-to-grapheme conversion mediated by dorsal tracts. Further research is required to elucidate the flexibility of spelling strategies and their adaptation to the specific orthographic and morphological characteristics of different writing systems.

## Supplementary Information

Below is the link to the electronic supplementary material.

Supplementary file1 (DOCX 2432 KB)

## Data Availability

Behavioral and imaging datasets analyzed during the current study are available from the corresponding author upon reasonable request. dMRI data were analyzed using open-source software, including mrDiffusion (https://github.com/vistalab/vistasoft), mrTrix3 (Tournier et al. 2019; https://www.mrtrix.org), and AFQ (Yeatman et al. 2012b; https://github.com/yeatmanlab/AFQ). Code and ROIs for the segmentation of the three SLF branches developed in the current study are available at https://github.com/yeatmanlab/AFQ/tree/master/SLF123.
