# Supplementary material for: White matter associations with spelling performance
Source: Brain Struct Funct. 2024 Mar 25;229(9):2115–35. doi: 10.1007/s00429-024-02775-7 (PMC11611966; doi:10.1007/s00429-024-02775-7)
Supplement: Supplementary file 1 — Supplementary file1 (DOCX 2432 KB) [file 429_2024_2775_MOESM1_ESM.docx]

**Supplementary Information**

**White matter associations with spelling performance**

Romi Sagi^1^ ^§^, J.S.H. Taylor^2^, Kyriaki Neophytou^3 5^, Tamar Cohen^1^, Brenda Rapp^3^, Kathleen Rastle^4^ and Michal Ben-Shachar^1, §^

^1^ The Gonda Multidisciplinary Brain Research Center, Bar-Ilan University, Ramat-Gan, Israel

^2^ Division of Psychology and Language Sciences, University College London, London, UK

^3^ Department of Cognitive Science, Johns Hopkins University, Baltimore, USA

^4^ Department of Psychology, Royal Holloway, University of London, UK

^5^ Department of Neurology, Johns Hopkins Medicine, Baltimore, USA

^§^ Corresponding authors: sagiromi@gmail.com; michalb@mail.biu.ac.il

| **Table S1**  **Spelling task items.** A selection of 40 words adapted from Burt & Tate (2002) with carrier sentences | | |
| --- | --- | --- |
| Sentence | Item | |
| The top student was very diligent | Diligent | |
| Friends have called him a hypocrite | Hypocrite | |
| There is a rebuttal to this point | Rebuttal | |
| The weapon could annihilate the city | Annihilate | |
| The word “apocalypse” has come to mean a major catastrophe | Apocalypse | |
| Bears are ravenous after hibernating | Ravenous | |
| She did not appreciate the subtlety of the argument | Subtlety | |
| The thief celebrated his acquittal | Acquittal | |
| The country was governed by a tyrannical regime | Tyrannical | |
| She had an insatiable thirst for stories about celebrities | Insatiable | |
| The response was succinct | Succinct | |
| His friends tried to dissuade him from flying | Dissuade | |
| The elderly lady received palliative care | Palliative | |
| After the musical key changed, there was a crescendo | Crescendo | |
| The aristocrat peered haughtily at his staff | Haughtily | |
| The police called the crime abhorrent | Abhorrent | |
| The singer was accompanied by the accordion | Accordion | |
| The phrase ‘downsizing’ is a euphemism for cuts | Euphemism | |
| She couldn’t keep up the masquerade | Masquerade | |
| The musician performed his repertoire | Repertoire | |
| The car had a hydraulic braking system | Hydraulic | |
| It would be sacrilege to spit in the communion cup | Sacrilege | |
| The toothpaste contained fluoride to reduce tooth decay | Fluoride | |
| Her stiletto heel hurt his foot | Stiletto | |
| The plate was made of porcelain | Porcelain | |
| His failures made him malevolent toward his peers | Malevolent | |
| The wall was covered in graffiti | Graffiti | |
| Dysentery is initially managed by maintaining fluid intake | Dysentery | |
| The empty bottle went into the recycling receptacle | Receptacle | |
| Running can aggravate pre-existing injuries | Aggravate | |
| The parties agreed to conciliate their dispute | Conciliate | |
| He had reached the pinnacle of his career | Pinnacle | |
| The accomplice in the murder was sent to prison | Accomplice | |
| One would have to be an imbecile to reject this offer | Imbecile | |
| During lent, one could practice abstinence | Abstinence | |
| Cartilage damage can result from a fall | Cartilage | |
| He was a bachelor and renowned misogynist | Misogynist | |
| The burglar was spotted by vigilant neighbours | Vigilant | |
| A bureaucrat is a person working for the government | Bureaucrat | |
| Eggs and chicken provide easily digestible protein | Digestible | |

| 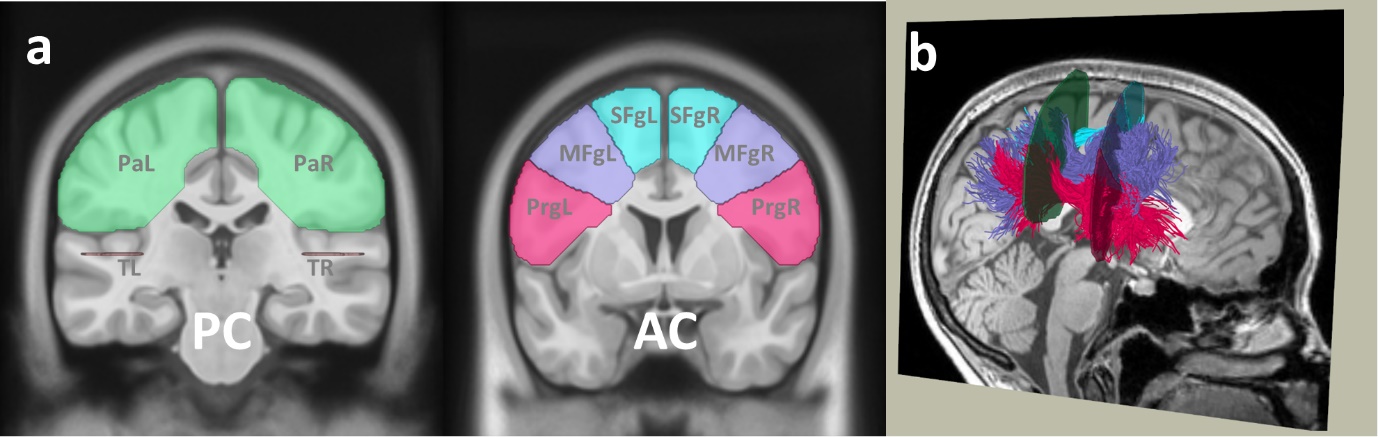 |
| --- |
| **Fig. S1 Automatic segmentation of the SLF three branches.** (a) ROIs, universally defined on a template, located in accordance with Thiebaut de Schotten et al. (2011). (b) An example from the right hemisphere of a single participant (female, 21), showing the segmentation of the SLF-I (in cyan), SLF-II (in purple), and SLF-III (in magenta) using CSD coupled with probabilistic tractography. Abbreviations: *Pa* parietal, *SF* superior frontal, *MF* middle frontal, *Prg* prefrontal gyrus, *T* temporal, *R* right hemisphere, *L* left hemisphere, *AC* anterior commissure, *PC* posterior commissure |

| **Table S2**  **Spelling and additional cognitive measures (*N* = 73).** See Methods, section 2.2.2 for detailed task descriptions | | | |
| --- | --- | --- | --- |
|  | *M* | *SD* | Range |
| Spelling (*accuracy*) | 0.41 | 0.234 | [0.075, 0.975] |
| Vocabulary (*accuracy*) | 0.78 | 0.089 | [0.575, 0.975] |
| Spoonerisms (*accuracy*) | 0.76 | 0.171 | [0.25, 1] |
| TOWRE PDE (*SS*) | 110.48 | 10.258 | [93, 130] |
| CToPP NW rep (*SS*) | 9.03 | 2.962 | [1, 15] |
| TOWRE SWE (*SS*) | 90.78 | 13.264 | [73, 130] |
| RAN letters (*s*) | 13.89 | 3.467 | [6.70, 24.76] |
| *TOWRE* Test of Word Reading Efficiency, *SWE* sight word efficiency, *PDE* phonemic decoding efficiency, *CToPP* Comprehensive Test of Phonological Processing, *NW rep* nonword repetition, *RAN* Rapid Automatized Naming, *SS* scaled scores, according to the age norms | | | |
|  | | | |


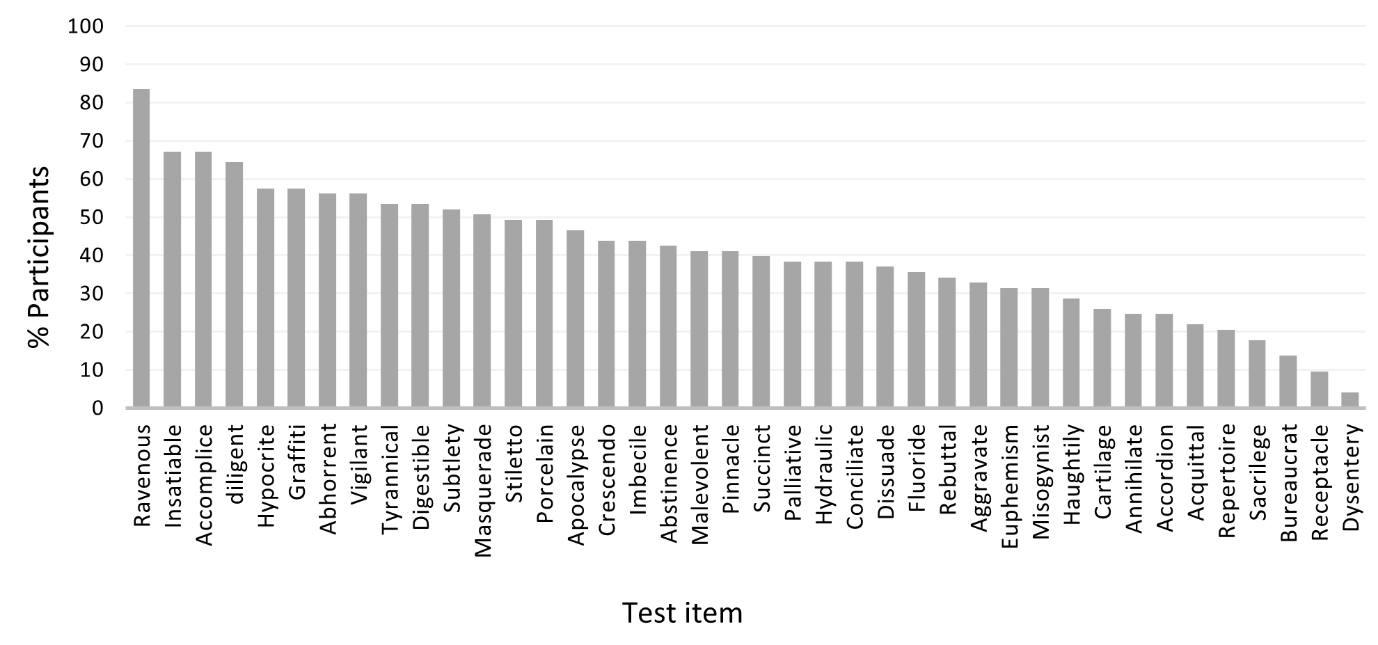


**Fig. S2 Accuracy per item.** Percentage of participants that responded correctly is shown for each item in the spelling task

| **Table S3**  **Differences between low-performing spellers (*n* = 41) and high-performing spellers (*n* = 32) on spelling and additional cognitive tasks.** See Methods, section 2.2.2 for detailed task descriptions | | | | |
| --- | --- | --- | --- | --- |
|  | Low-performing spellers | High-performing spellers | *t* (*df*) | *p* |
|  | *M* (*SD*)  [Range] | *M* (*SD*)  [Range] |  |  |
| Spelling (*accuracy*) | 0.23 (0.094)  [0.075, 0.425] | 0.63 (0.158)  [0.45, 0.975] | 13.3 (71) | <.0001 * |
| Vocabulary (*accuracy*) | 0.74 (0.077)  [0.575, 0.9] | 0.84 (0.071)  [0.7, 0.975] | 5.7 (71) | <.0001 * |
| Spoonerisms (*accuracy*) | 0.71 (0.187)  [0.25, 1] | 0.83 (0.122)  [0.6, 1] | 3.1 (71) | <.01 * |
| TOWRE PDE (*SS*) | 107.0 (9.767)  [93, 130] | 114.94 (9.207)  [99, 130] | 3.5 (71) | <.001 * |
| CToPP NW rep (*SS*) | 8.49 (2.675)  [3, 14] | 9.72 (3.205)  [1, 15] | 1.8 (71) | .079 |
| TOWRE SWE (*SS*) | 102.76 (13.187)  [80, 130] | 101.94 (13.560)  [73, 130] | -0.3 (71) | .80 |
| RAN letters  (*s*) | 13.95 (3.396)  [7.78, 24.76] | 13.83 (3.608)  [6.70, 24.41] | -0.1 (71) | .88 |
| * Significant group difference, controlling the FDR at 5%. *TOWRE* Test of Word Reading Efficiency, *SWE* sight word efficiency, *PDE* phonemic decoding efficiency, *CToPP* Comprehensive Test of Phonological Processing, *NW rep* nonword repetition, *RAN* Rapid Automatized Naming, *SS* scaled scores, according to the age norms | | | | |

| **Table S4**  **Evaluating the contribution of mean tract-FA to spelling accuracy scores (*N* = 73).** Using a forward and backward stepwise linear regression model across the full sample, out of the 10 tracts of interest, the left ILF and the right SLF-III were identified as significant predictors of the spelling accuracy scores. At each step, thresholds to add or remove variables were set to *p* < .05 (*p*-Enter) and *p* > .1 (*p*-Remove). Overall regression: R^2^ = .16, *F*(2, 69) = 6.6, *p* = .0023 | | | | | |
| --- | --- | --- | --- | --- | --- |
| Predictor | B^1^ | SE^1^ | β^2^ | *t* | *p* |
| (Constant) | -0.10 | 0.58 |  | -0.17 | 0.8643 |
| Left ILF | 3.31 | 1.02 | 0.37 | 3.23 | 0.0019 |
| Right SLF-III | -2.29 | 0.96 | -0.27 | -2.37 | 0.0205 |
| ^1^ Unstandardized Coefficients, ^2^ Standardized Coefficient | | | | | |

|  |
| --- |
|  |
|  |
| **Fig. S3 Tract-FA not predictive of selling accuracy scores of the: SLF-I (left - a,b; right - c,d), SLF-II (left - e,f; right - g,h), and arcuate (left - i,j; right - k,l).** Tractograms depict the tracts in a single participant, with white dashed lines marking the ROIs between which FA values were averaged. Scatter plots show the covariation between spelling scores and tract-FA across participants (*N* = 73). r values are Spearman’s correlation coefficients |


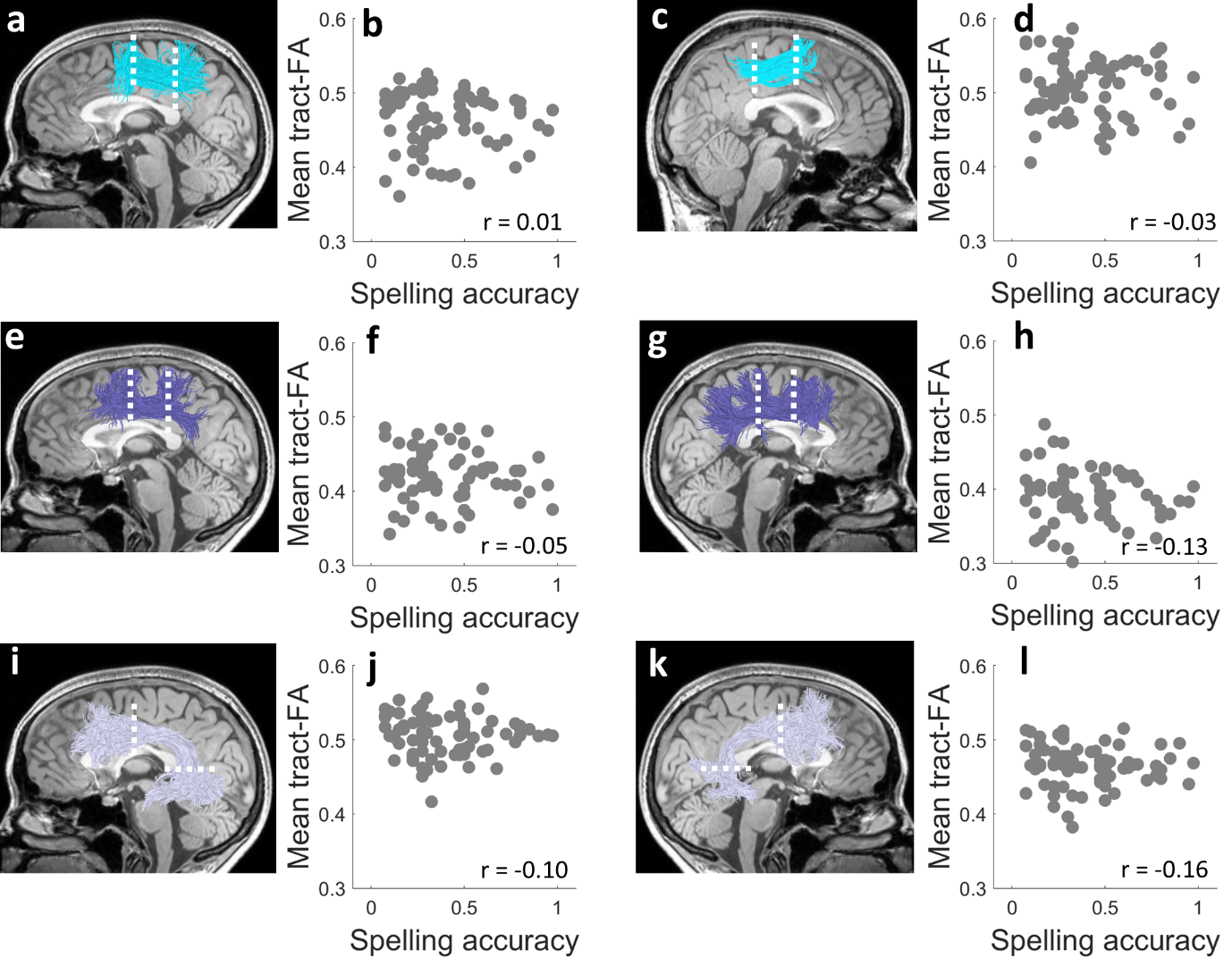


| 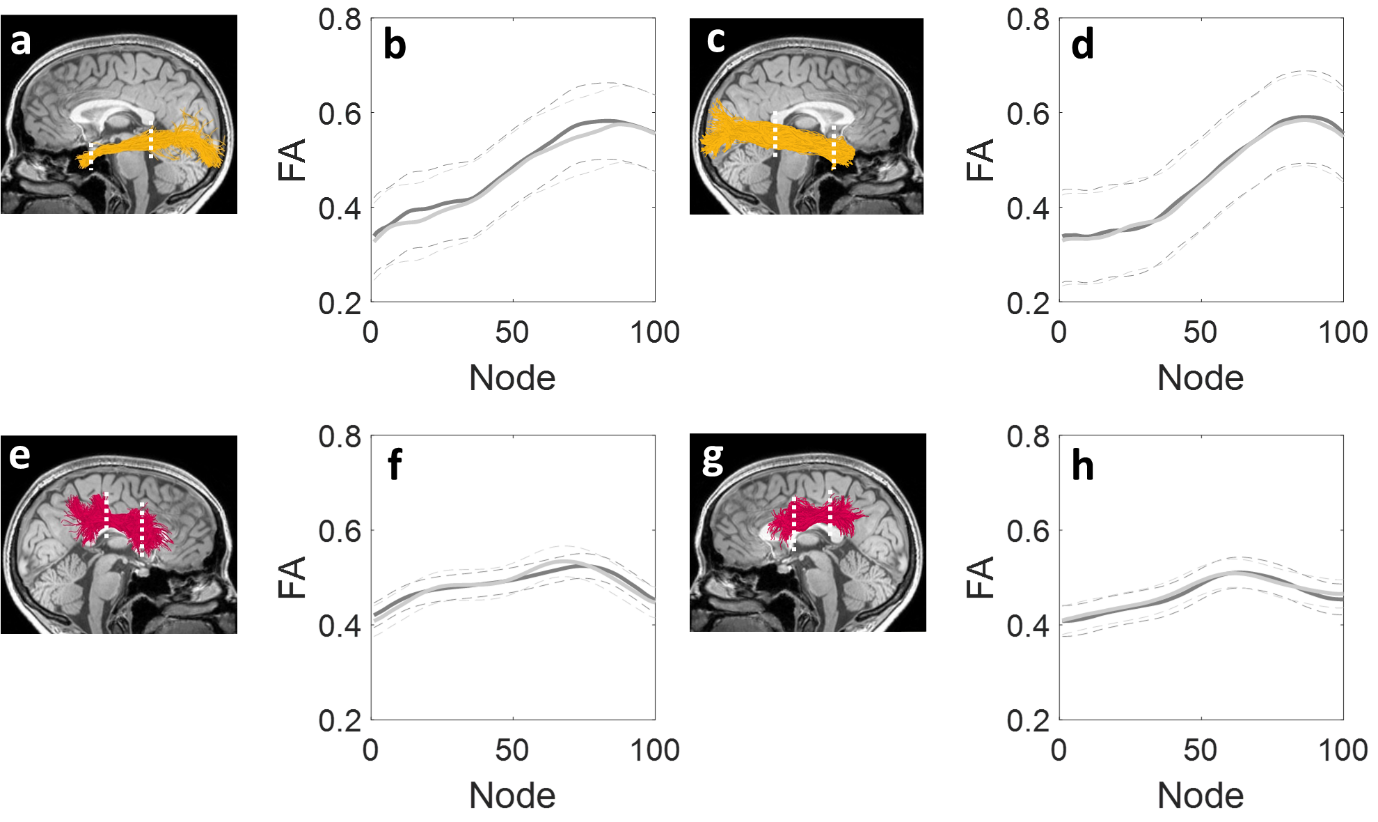 |
| --- |
| **Fig. S4 No group differences in FA profiles.** FA profiles are shown for the left ILF (a-b), right ILF (c-d), left SLF-III (e-f), and right SLF-III (g-h), averaged across high-performing spellers (dark gray line) and low-performing spellers (light gray line). The profiles are plotted along 100 equidistant nodes between two waypoint-ROIs from anterior (node 0) to posterior (node 100). Dashed lines denote ±1 standard error of the means for each group. Tractograms demonstrate each tract in a single participant, with the locations of the ROIs, marked with white dashed lines |

| **Fig. S5 Individual orthographic and phonological distances.** Each circle indicates the mean distance score of an individual’s errors (see Methods, section 2.3.2 for the calculation of orthographic and phonological distances). The central mark denotes the median, and the bottom and top edges of the box indicate the 25th and 75th percentile, respectively | 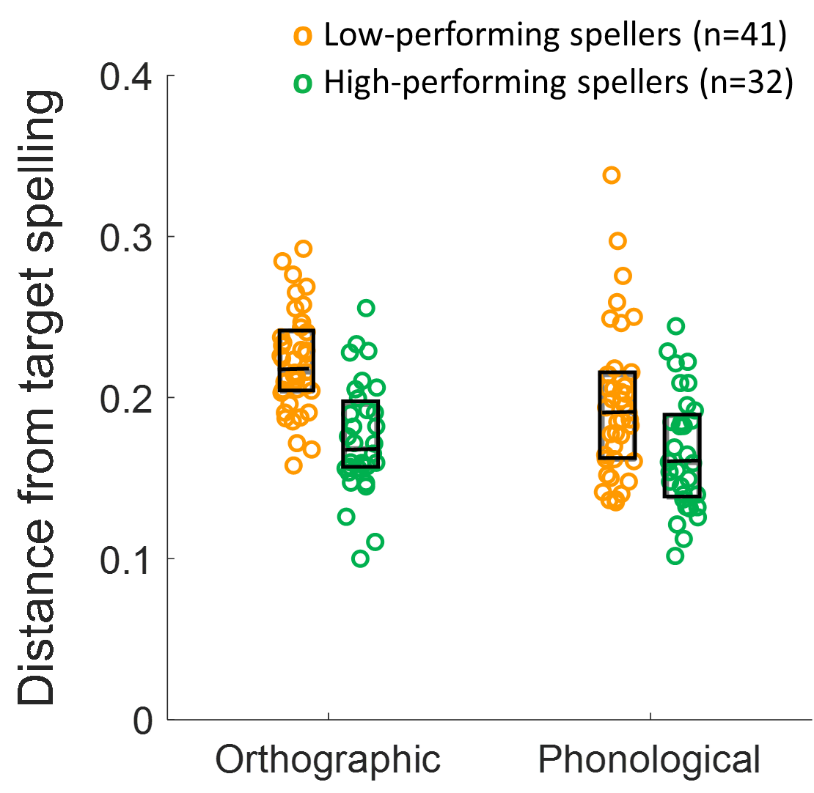 |
| --- | --- |

| 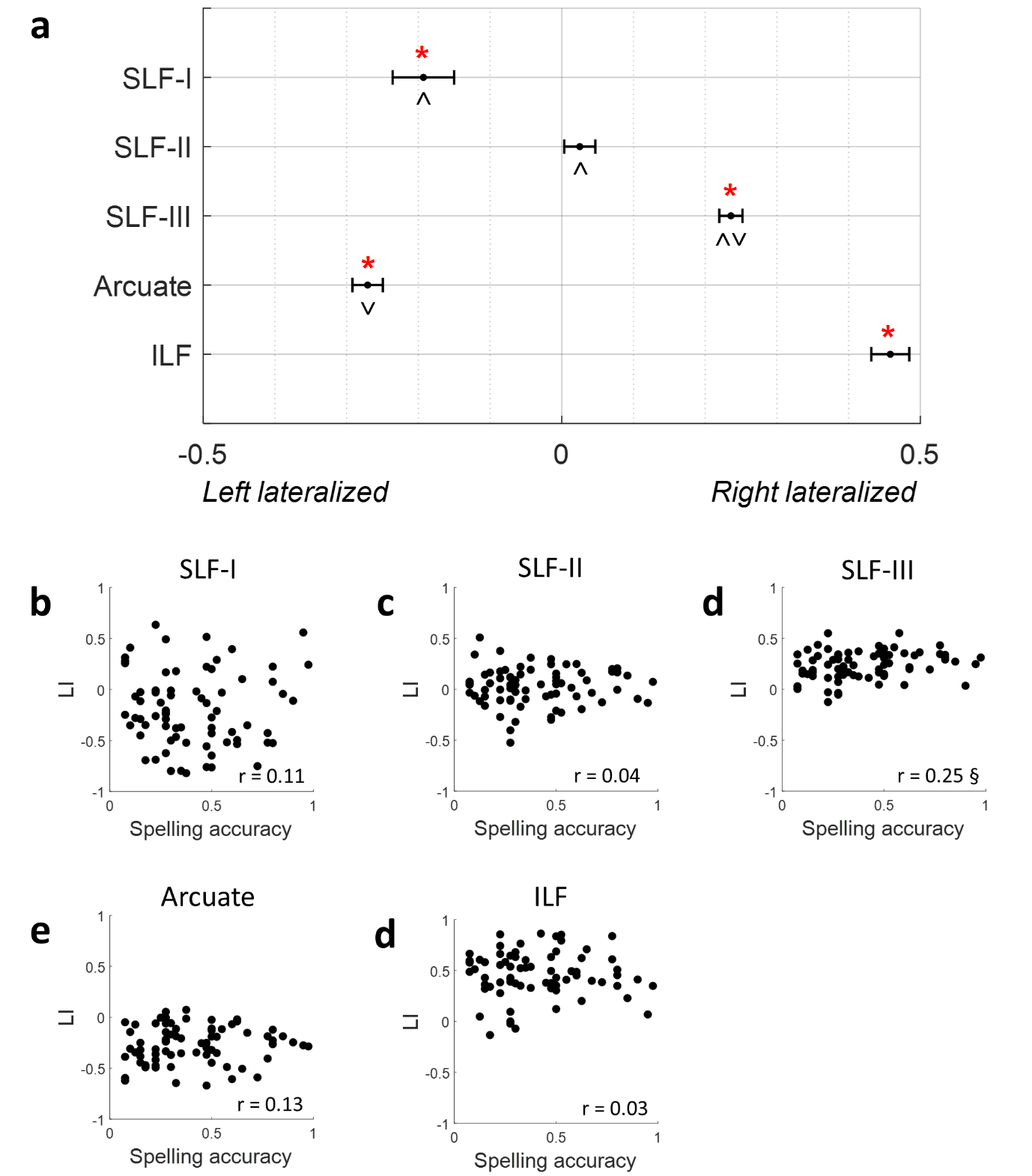 |
| --- |
| **Fig. S6 Lateralization results.** (a) Mean LIs calculated for the number of streamlines in each pair of homologous tracts. Error bars indicate ±1 standard error of the means. * indicates significant right-left asymmetry, Bonferroni corrected across five comparisons (*p* < .01). ˄ indicates consistent patterns of tract lateralization with Amemiya et al. (2021), ˅ indicates consistency with Thiebaut de Schotten et al. (2011b). Scatter plots depict the associations between individuals’ spelling scores and LIs in the SLF-I (b), SLF-II (c), SLF-III (d), arcuate (e), and ILF (d). r values are Spearman’s correlations. § *p* < .05, uncorrected across five comparisons |
